# Effects of SiO_2_, ZrO_2_, and BaSO_4_ nanomaterials with or without surface functionalization upon 28-day oral exposure to rats

**DOI:** 10.1007/s00204-014-1337-0

**Published:** 2014-08-28

**Authors:** Roland Buesen, Robert Landsiedel, Ursula G. Sauer, Wendel Wohlleben, Sibylle Groeters, Volker Strauss, Hennicke Kamp, Bennard van Ravenzwaay

**Affiliations:** 1 Experimental Toxicology and Ecology, BASF SE, 67056 Ludwigshafen am Rhein, Germany; 2 Scientific Consultancy - Animal Welfare, 85579 Neubiberg, Germany; 3 Polymer Physics, BASF SE, 67056 Ludwigshafen am Rhein, Germany

**Keywords:** Nanomaterial (NM), Surface functionalization, Oral route of exposure, Subacute toxicity, OECD test guideline 407, Metabolomics

## Abstract

The effects of seven nanomaterials (four amorphous silicon dioxides with or without surface functionalization, two surface-functionalized zirconium dioxides, and barium sulfate) upon 28-day oral exposure to male or female rats were investigated. The studies were performed as limit tests in accordance with OECD Test Guideline 407 applying 1,000 mg test substance/kg body weight/day. Additionally, the acute phase proteins haptoglobin and α2-macroglobulin as well as cardiac troponin I were determined, and metabolome analysis was performed in plasma samples. There were no test substance-related adverse effects for any of the seven nanomaterials. Moreover, metabolomics changes were below the threshold of effects. Since test substance organ burden was not analyzed, it was not possible to establish whether the lack of findings related to the absence of systemic exposure of the tested nanomaterials or if the substances are devoid of any potential for toxicity. The few published subacute oral or short-term inhalation studies investigating comparable nanomaterials (SiO_2_, ZrO_2_, and BaSO_4_) also do not report the occurrence of pronounced treatment-related findings. Overall, the results of the present survey provide a first indication that the tested nanomaterials neither cause local nor systemic effects upon subacute oral administration under the selected experimental conditions. Further investigations should aim at elucidating the extent of gastrointestinal absorption of surface-functionalized nanomaterials.

**Electronic supplementary material:**

The online version of this article (doi:10.1007/s00204-014-1337-0) contains supplementary material, which is available to authorized users.

## Introduction

Due to the well-known effects of generic particulate dusts on the human lung and its thin air–blood barrier, uptake by inhalation is expected to be the critical route of exposure for most nanomaterials (NMs) (Hankin et al. 2011). In recognition thereof, information on in vitro and in vivo pulmonary effects of NMs is increasingly becoming available (Landsiedel et al. 2012a). However, consumers might also be exposed to NMs orally, e.g., when they are taken up as additives to food products, upon release from food contact materials, or when they are contained in cosmetic articles, such as lipstick or toothpaste. Furthermore, NMs may be taken up orally, when they are used in the pharmacological context, e.g., to increase oral drug bioavailability or to promote applicability of drugs (Bouwmeester et al. 2011; Fröhlich and Roblegg 2012). Finally, also inhaled NMs may be translocated into the pharynx by the mucociliary clearance system of the respiratory tract and be swallowed into the gastrointestinal tract (Landsiedel et al. 2012b).

Upon ingestion, NMs can be taken up into the body. It is generally agreed that particle absorption in the gastrointestinal tract is low, but increases with decreasing particle diameter (Florence 2005; Landsiedel et al. 2012b). Further, factors affecting oral NM uptake include particle surface charge, hydrophobicity, and the presence or absence of surface ligands (Florence 1997). Large inter-individual differences in NM uptake have been observed, and differences in the diet, in mucus secretion and composition, in pH values, in gastrointestinal transit time, and in gastrointestinal flora all have been recognized to influence NM uptake (Fröhlich and Roblegg 2012). In the intestine, the microfold cells (M cells) in the Peyer’s patches are important sites of NM uptake, but the enterocytes, the intestinal epithelial cells, also play an active role in particle translocation across the intestinal wall (Delie et al. 1998; Hillyer and Albrecht 2001).

Oral uptake of nanoparticular food additives or ingredients of cosmetic articles, etc., by consumers is likely to occur at low doses over long periods of time. Nevertheless, to date, only few reports on in vivo effects of NMs upon subacute or sub-chronic oral exposure to rodents are available (EFSA 2009; Dekkers et al. 2013). The majority of these investigations address the effects of silver nanoparticles, but different metal, metal oxide and carbon-based NMs were also evaluated, some of which causing histopathological changes, e.g., in the liver or kidneys, or alterations in blood parameters (Fröhlich and Roblegg 2012; van der Zande et al. 2012; Hadrup et al. 2012a; Dekkers et al. 2013). The limited number of oral repeated-dose toxicity studies in rodents—and oftentimes the lack of standardization of substance characterization and testing methods, however, does not allow a final conclusion on the effects that different NMs might cause upon subacute or sub-chronic oral administration.

Against this background, in the present study, the effects of a variety of different NMs upon 28-day oral exposure to rats were investigated. Due to their relevance for the oral route of exposure, different silicon dioxide (SiO_2_) and zirconium dioxide (ZrO_2_) NMs were selected for the oral toxicity studies, and they were compared to nanoparticular barium sulfate (BaSO_4_), for which extensive physico-chemical information is already available since it is the OECD reference nanomaterial NM-220 (OECD 2008a; Hellack et al. 2012).

Amorphous SiO_2_ NMs are produced in annual volumes of megatons and have universal applications. As food additives, they are used, e.g., as anticaking agents (such as ‘E551’) to prevent the clumping of powdered food products or to clarify liquids. Therefore, SiO_2_ NMs are especially relevant for oral uptake (Anon 2012; Wohlleben et al. 2014). ZrO_2_ is a highly engineered nanomaterial, which is crystalline, instead of amorphous. It further has smaller particles than SiO_2_, with a size distribution [determined by transmission electron microscopy (TEM)] down to 3 nm. Hence, testing SiO_2_ and ZrO_2_ NMs under the same experimental conditions allowed assessing possible size-dependent NM effects. Consumer-relevant applications of ZrO_2_ NMs are in self-cleaning coatings in stoves or other ceramic surfaces, and also in dental fillings or prostheses (Anon 2012; Hellack et al. 2012). Therefore, the oral route of exposure is also relevant for ZrO_2_ NMs.

Also the OECD reference material BaSO_4_ NM-220 can be taken up orally: Micron-sized BaSO_4_ occurs can be ingested, and it is used as a radio-contrast agent, whereas the application of nanosized BaSO_4_ as an additive to dental cements or medicinal product materials is currently under investigation (Ricker et al. 2008; Aninwene et al. 2013).

To analyze the influence of physico-chemical characteristics of NMs on their biological effects in vivo (Wohlleben et al. 2014), the SiO_2_ and ZrO_2_ NMs were selected having different, systematically varied surface functionalizations. In in vitro NM toxicology, NM charge and protein affinity (i.e., corona formation) are reported to affect the toxicity of otherwise identical substances. In order to test whether this hypothesis also holds true for in vivo toxicity, the functionalizing agents were chosen to span the widest possible range of surface properties. Thereby, it was investigated how changes in NM structure affect their activity in biological systems, a necessary prerequisite to establishing structure–activity relationships (Wohlleben et al. 2014).

Based on these considerations, the following surface modifications were chosen: SiO_2_ NM was tested with negative (SiO_2·_phosphate), neutral [SiO_2·_polyethyleneglycol (SiO_2·_PEG)] and slightly positive (SiO_2·_amino) charges. ZrO_2·_TODS [with trioxadecanoic acid (TODS) surface modification] had the same slightly positive electrical charge as SiO_2·_amino. With their short-chain functionalizations, both of these substances were free of steric functionality. ZrO_2·_acrylate was strongly negative, thereby additionally introducing steric stabilization (oligomeric functionalization) that was found to effectively reduce corona formation (Wohlleben et al. 2014). The surface functionalization of the NMs and their delivery as dispersions, instead of powders, further served to prevent nanoparticle agglomeration optimizing their nanoform applicability in biological studies (Hellack et al. 2012). Additionally, SiO_2_ NM was tested without surface functionalization, whereas ‘naked’ ZrO_2·_NMs that had already been assessed as being non-toxic in earlier in vitro and inhalation toxicity studies (Kuhlbusch et al. 2009) was not included in the present study.

The subacute oral toxicity studies were performed as limit tests in accordance with OECD test guideline (TG) 407 (OECD 2008b), since, from assessment of previous short-term inhalation studies (Landsiedel et al. 2014), no effects were expected at daily dosages of 1,000 mg/kg body weight. In addition to the basic hematological and clinical chemistry parameters listed in OECD TG 407, the acute phase proteins haptoglobin and α2-macroglobulin as well as cardiac troponin I were determined. Such biomarkers have been suggested as useful in detecting nanoparticle-induced effects at very early stages preceding clinically observable effects (Higashisaka et al. 2011; Nagano et al. 2012).

Additionally, metabolomics or ‘metabolite profiling’ based upon gas chromatography–mass spectrometry (GC–MS) and liquid chromatography–mass spectrometry/mass spectrometry (LC–MS/MS) was performed in plasma samples (van Ravenzwaay et al. 2010a; Kamp et al. 2012a, b). Metabolomic investigations have been established as useful tools to recognize early toxicological effects, already at stages preceding clinically observable findings. Metabolite level changes can be interpreted biochemically and by comparing alterations with those of about 500 reference compounds in the MetaMap^®^Tox database, thereby revealing possible toxicological modes of action (MoAs, i.e., the specific biochemical interactions through which a substance produces its influence on key processes in living organisms; van Ravenzwaay et al. 2007, 2010a, b, 2012a, b; ECETOC 2010; Kamp et al. 2010). Since changes are detectable in body fluids, such as urine and plasma, metabolomics have the potential to reduce and refine animal testing. Furthermore, metabolomics might help bridging in vitro testing to in vivo relevance (ECETOC 2010), thereby possibly also contributing to replacing animal testing also for NM toxicity assessment.

In evaluating findings, special attention was paid to ensuring their consistent and transparent interpretation. To differentiate between adverse and non-adverse effects, the structured approach proposed by the European Centre for Ecotoxicology and Toxicology of Chemicals (ECETOC 2002) was applied. To recognize adverse effects, the toxicologist first has to apply defined criteria to decide whether differences from control values are treatment-related effects or occur by chance, i.e., incidentally. In a second step, again applying defined criteria, only those differences judged to be treatment-related are evaluated further to discriminate between those that are adverse and those that are not (ECETOC 2002).

## Materials and methods

### Test substances and particle characterization

The following seven test substances were selected from the set of nanoGEM test substances (Hellack et al. 2012; Wohlleben et al. 2014):

- SiO_2·_naked: SiO_2_ without surface modification (Levasil^®^ 200);
- SiO_2·_PEG: Levasil^®^ 200 with covalent surface functionalization with a low-molar-mass silane having a PEG end group with a molecular weight of 500 g/mol (PEG-500), imparting some steric stabilization;
- SiO_2·_phosphate: Levasil^®^ 200 with covalent surface functionalization with a low-molar-mass silane having a negatively charged phosphonate end group on a flexible, short C3-linker;
- SiO_2·_amino: Levasil^®^ 200 with covalent surface functionalization with a low-molar-mass silane having a positively charged amino end group on the same C3-linker;
- ZrO_2·_acrylate: ZrO_2_ with acrylate surface modification having a strongly negative electrical charge and imparting steric stabilization;
- ZrO_2·_TODS: ZrO_2_ with trioxadecanoic acid (TODS) surface modification having a slightly positive electrical charge;
- BaSO_4_: reference material NM-220 from the OECD Working Party for Manufactured Nanomaterials (WPMN) Sponsorship Programme for the Testing of Manufactured Nanomaterials (OECD 2008a and 2010; *cf.* http://ihcp.jrc.ec.europa.eu/our_activities/nanotechnology/nanomaterials-repository).

The SiO_2_ NMs were supplied by BASF SE, Ludwigshafen, Germany; the ZrO_2_ NMs by Ceranovis AG, Saarbrücken, Germany; and BaSO_4_ NM-220 by Solvay, Brussels, Belgium. All test substances were delivered as dispersions, except for BaSO_4_, which was delivered as powder. All test substances were characterized in detail in accordance with the physico-chemical endpoints described in the guidance on information requirements for nanomaterials (ECHA 2012) to EU regulation No. 1907/2006 on the Registration, Evaluation, Authorisation and Restriction of Chemicals (REACH; Anon 2006). The following test substance properties were determined making use of the indicated methodologies (Hellack et al. 2012; Wohlleben et al. 2013; Landsiedel et al. 2014):

Mean primary particle size and primary particle size (PPS) distribution (TEM); hydrodynamic particle size in water [dynamic light scattering (DLS) and analytical ultracentrifugation (AUC)]; particle morphology [light microscopy and scanning electron microscopy (SEM)]; crystallinity [X-ray diffraction (XRD)] surface chemistry, purity, and crystalline phase [X-ray photoelectron spectroscopy (XPS)]; organic surface functionalization, [secondary ion mass spectrometry (SIMS)]; iso-electric point and zeta-potential (electrophoretic mobility titration); surface reactivity and radical formation potential (Electron spin resonance (ESR) making use of centrophenoxine (CPH) or dimethyl-pyrroline-N-oxide (DMPO) spin traps).

For three sample substances, i.e., SiO_2·_naked, SiO_2·_PEG, and ZrO_2·_TODS, the state of agglomeration in the test substance vehicle of the 28-day oral toxicity studies, i.e., phosphate buffered saline (PBS) supplemented with 1 g/L bovine serum albumin (BSA; in the following: ‘PBS + BSA’), was determined by laser diffraction and AUC.

Due to limited test substance availability, the other nanomaterials could not be assessed in the vehicle of the present study. However, for all test substances, dispersability in water or Dulbecco’s modified Eagle medium (DMEM) supplemented with 10 % fetal calf serum (FCS; in the following ‘DMEM + FCS’) were determined by AUC (Wohlleben et al. 2012, 2013; Landsiedel et al. 2014). DMEM + FCS represents a biological medium that is comparable to the test substance vehicle. Therefore, NM dispersability is expected to be comparable in DMEM + FCS and PBS + BSA, and this data are included in Table 1 even though DMEM + FCS was not used as vehicle in the oral studies.

**Table 1:** Physico-chemical characterization of the test substances (adapted from: Hellack et al. 2012; Wohlleben et al. 2013)

Property	Method	Units	SiO_2·_naked	SiO_2·_PEG	SiO_2·_amino	SiO_2·_phosphate	ZrO_2·_acrylate	ZrO_2·_TODS	BaSO_4_ NM-220
Form of delivery		Weight %	Suspension (40 %)	Suspension (20 %)	Suspension (20 %)	Suspension (20 %)	Suspension (35 %)	Suspension (45 %)	Powder (purity: >90 %)
Intended surface functionalization	Educts in synthesis	Qualitative	None	PEG-500	aminopropyl trimethoxy silane	Triphenyl methyl-phosphonium	Acrylic acid	Trioxadecanoic acid	Undisclosed organics
Particle morphology	LM/SEM	Qualitative	Uniform, globular	Uniform, globular	Uniform, globular	Uniform, globular	Irregular, globular	Irregular, globular	Globular, few cubic or rod-shapes, agglomeration
Crystallinity	XRD	Qualitative	Amorphous	Amorphous	Amorphous	Amorphous	Amorphous, monocline, and tetragonal	Amorphous, monocline, and tetragonal	Crystalline, orthorombic
Primary particle size (mean)	TEM	nm	15	15	15	15	9	9	32
Primary particle size distribution	TEM	nm	5–50	8–45	5–50	5–50	9	3–15	10–150
Particle size (H_2_O)	DLS D_50_ (H_2_O)	nm	40	50	42	40	9	9	350
Particle size/dispersability (H_2_O)	AUC D_50_ (H_2_O)/AAN	nm/unitless	19/1	21/1	20/1	20/1	27/3	11/1	350/11
Particle size/dispersability in DMEM/FCS	AUC (D_50_/AAN)	nm/unitless	420/28	3,200/213	1,350/90	30/2	315/32	860/86	285/9
Particle size/dispersability in PBS + BSA (pH value: 7.4)	AUC (D_50_/non-adsorbed albumin)	nm/%	15/non-adsorbed albumin: 42 %	29,000/non-adsorbed albumin: 41 %	Not determined	Not determined	Not determined	17,000/non-adsorbed albumin: 38 %	Not determined
Surface composition	XPS/supported by SIMS	Atom-%/qualitative	Si: 29;O: 66; C: 4 (C–C, C–H, C–O, C=O); Na: 1	Not determined/confirmed PEG- (CH_2_CH_2_O)	Not determined/confirmed AMEO	Si: 29; O: 66; C: 4.6; Na: 0.5 (P, N not detected)/confirmed PO_2_, PO_3_	Zr: 23; O: 58; C: 19/confirmed acrylic acid	Zr: 24; O: 63; C: 11; N: 0.7; S: 0.2/confirmed organic acid	Ba: 13; O: 52; C: 17; S: 11; Cl: 3; P: 3; N: 1
Iso-electric point	Electrophoretic mobility titration	pH	<1	4	7.2	<1	<1	7.1	3.3
Zeta-potential at pH 7.4		mV	−39	−26	0	−42.9	−39	−6.5	−39
Surface reactivity*	ESR/CPH spin trap	Relative to D_2_O	4/ p-f s: 0.88	1/ p-f s: 3.4	1/ p-f s: 1.1	2.2/ p-f s: 1.2	1/ p-f s: 2	0.54/ p-f s: 5.7	2
Formation of OH radicals (ROS)*	ESR/DMPO spin trap	Relative to D_2_O	11/ p-f s: 6.3	11/ p-f s: 13	21/ p-f s: 5.2	19/ p-f s: 5	3.6/ p-f s: 1.5	0.94/ p-f s: 1.3	2

AAN: average agglomeration number derived from the ratio of the volume-based median particle size to the average equivalent spherical volume derived from the BET gas adsorption (ECHA 2012)

*ROS* reactive oxygen species

*p-f s* particle-free supernatant

* Surface reactivity and formation of reactive oxygen species (ROS) are determined relative to the ‘reference material’ deuterium oxide (D_2_O; ^2^H_2_O). Assuming a 30 % variability of the methodology, only measurements >1.3 are considered relevant. Of note, this value should only serve as a guiding principle, and not as an absolute value

In addition to the parameters listed in Table 1, for BaSO_4_, the specific surface area (SSA) was determined to be 41 m^2^/g, making use of the method of Brunauer–Emmett–Teller (BET). All other test substances are not submitted to the BET method, since they are supplied as suspensions and the SSA is determined in the material’s dry state. For all four SiO_2_ NMs, the SSA of 200 m^2^/g indicated by the supplier of Levasil^®^ 200 was confirmed indirectly by TEM. Since the functionalization agents only partially covered the surface of the well-dispersed primary particles, the effective specific surface is not expected to change due to surface functionalization. Functionalization was confirmed by SIMS spectra, while no shells are detected by TEM

### Preparation of test substances

The original test suspensions, as provided by the suppliers, were shaken and mixed for 2 min using a vortex mixer to ensure a homogeneous distribution of particles. Next, the desired amount of test substance was weighed and then filled up with the test substance vehicle PBS + BSA to obtain uniform test substance solutions of 10 wt% solutions. Test substance preparations were produced daily and were kept homogenous until administration by continuous stirring with a magnetic stirrer.

Since NMs can agglomerate and sediment quickly in suspensions and this can considerably affect the final, effective dosage reaching the target organism, it is essential to assess the homogeneity of test substances and to verify the effective concentration in the test substance preparations. Therefore, at the onset of the administration period, homogeneity and concentration control analyses of all test substance suspensions (‘as delivered’ and ‘as prepared’ in PBS + BSA) were performed by inductively coupled plasma—optical emission spectrometry (ICP–OES). For this purpose, three separate samples of the test substance preparations were taken from the bottom, middle, and top layers of the vials (which would necessarily have the same test substance concentrations in homogenous suspensions).

The content of the metallic element of the respective test substances (i.e., silicon in the case of the SiO_2_ NMs, zirconium in the case of the ZrO_2_ NMs, and barium for BaSO_4_) was measured and the mass of the entire test substance molecule derived from these measurements. The mass of the substances used for surface functionalization was considered to be negligible (RIP-oN 1 2011).

### Study performance

The 28-day oral toxicity studies were performed with male and female Wistar Crl:WI(Han) rats (Charles River Laboratories, Sulzfeld, Germany) following a five- or nine-day acclimatization period. The animals were 42 ± 1 days of age at the onset of the study and free from clinical signs. The animal facility that all animal work was performed in holds a certificate from the International Association for Assessment and Accreditation of Laboratory Animal Care (AAALAC). The rats were housed in groups of 5 animals in polysulfonate cages (Tecniplast^®^, Hohenpeißenberg, Germany; floor area approximately 2,065 cm^2^) with dust-free wooden bedding. Wooden gnawing blocks (Type NGM E-022; Abedd^®^ Lab. & Vet. Service GmbH, Vienna, Austria) were provided to the animals for environmental enrichment. The animal studies were performed with approval of the local regulatory agencies, and all study protocols complied with the federal guidelines.

The experiments were performed as limit tests in accordance with OECD TG 407, applying uniform test substance dosages of 1,000 mg/kg body weight (bw)/day (*cf.* paragraph 18 of OECD TG 407). The test substance preparations were administered daily by gavage over a period of 4 weeks to groups of 5 male and 5 female rats. For logistical reasons, the study had to be conducted in two separate sub-studies (albeit under otherwise identical experimental conditions), with SiO_2·_PEG, SiO_2·_phosphate, SiO_2·_amino, ZrO_2·_acrylate, and ZrO_2·_TODS tested in the first study (in the following ‘sub-study A’) and SiO_2·_naked and BaSO_4_ NM-220 tested in the second study (‘sub-study B’). In each sub-study, control groups of 5 male and 5 female rats, each, received only the vehicle PBS + BSA. According to Gad et al. (2006) and in-house experience, the test substance vehicle PBS is well tolerated by rats at dosages of 10 mL/kg body weight over administration periods of up to 1 month.

Details on the performance of the Detailed Clinical Observations, regular health inspections, assessment of food and water consumption and the determination of the body weight are provided in the Supplementary Information (SI).

Hematological and clinical chemical examinations as well as urinalyses were performed toward the end of the administration period, and all standard parameters listed in OECD TG 407, paragraphs 32, 34, and 35, were evaluated for all animals. Additionally, the acute phase proteins haptoglobin and α2-macroglobulin were determined in serum samples from all animals and troponin I in serum samples from the animals used in sub-study A. Further details on the blood sampling and assessment of hematological parameters, clinical chemistry, and urinalysis are provided in the SI.

In evaluating the blood parameter results from the test groups, the data were compared not only to the corresponding data recorded for the control groups, but also to historical control data from unpublished in-house studies (except for the parameter ‘acute phase proteins’ for female rats, for which no historical control data were available; *cf.* SI for details on the unpublished in-house data).

Upon completion of the administration period, all rats were killed by decapitation under isoflurane anesthesia after food withdrawal for at least 16 h (except for the one female animal of the SiO_2·_naked test group that died due to gavage error). The exsanguinated animals were subjected to a full, detailed gross necropsy assessing and weighing all organs listed in OECD TG 407, paragraph 40. Additionally, all organs listed in OECD TG 407, paragraph 43, were preserved in neutral-buffered 10 % formalin (NBF) or modified Davidson’s solution for histopathological examination. After paraplast embedding, the paraplast blocks were cut with 2–3-µm thickness, cuts were mounted on glass slides and stained with hematoxylin and eosin (H&E; Merck, Darmstadt, Germany) for light microscopic assessment. As appropriate, any findings were either marked as being ‘present’ or they were graded (1–5) with increasing severity (minimal to massive), increasing numbers of affected units, etc. (very few to extensive numbers), or increasing size of affected tissue, etc. (very small to extensive size). All histopathological assessments were performed by a well-experienced board-certified (DECVP) veterinarian toxicopathologist.

### Metabolome analysis with MetaMap^®^Tox methodology

As described by van Ravenzwaay et al. (2007) and Kamp et al. (2012a), EDTA-K_3_ blood samples of all rats taken on study day 28 were analyzed in regard to their metabolite profiles upon metabolite extraction by a proprietary method: GC–MS and LC–MS/MS were applied for broad profiling and hormone measurement. The method resulted in 225 semi-quantitative analytes, 171 of which are chemically identified and 54 are structurally unknown. Analysis of the recorded metabolite profiles was performed making use of the MetaMap^®^Tox database (van Ravenzwaay et al. 2012a; *cf.* Information box MetaMap^®^Tox methodology).

**Table:** Information box: MetaMap^®^Tox methodology

The MetaMap^®^Tox database encompasses the metabolome profiles from rat plasma for approximately 500 pharmaceuticals, chemicals, and agrochemicals. These data had been determined by a special 28-day study protocol including control groups and low- and high-dose test groups of 5 male and 5 female Crl:WI(Han) rats, each, and plasma sampling after 7-, 14-, and 28-day test substance exposure. Discriminating metabolite patterns for various toxicological MoAs have been developed based on the common metabolome changes induced by (as a rule) at least three different chemicals included in the MetaMap^®^Tox database that share a common toxicological MoA (i.e., the reference compounds). Iteratively, an expert panel of experienced toxicologists modifies the list of metabolites in order to obtain sufficient sensitivity and selectivity against the reference data in the database.
During the evaluation of blood samples, the pattern ranking itself is a two-step process. First, applying a median r value metric, an algorithm used in the database yields a ranking list that is based on similarity of the metabolic profile of the test compound in comparison with the specific patterns listed in MetaMap^®^Tox. Second, the expert panel of experienced toxicologists evaluates the metabolite changes to determine which pattern matches might constitute ‘confirmed’ matches. In the course of this evaluation, the number of consistently changed metabolites as well as quality and importance of the metabolite changes for a given toxicological MoA is taken into consideration.
The European Centre for Ecotoxicology and Toxicology of Chemicals has defined criteria to determine adverse effects based on ‘-omics’ data (ECETOC 2008, 2010, 2013). According to these criteria, an adverse effect has to be based on, firstly, a significant overall effect within the data set and, secondly, the presence of changes that can be attributed to an adverse and clinically relevant phenotypical effect, either on the basis of biological pathway information or by comparison with reference data, which are predictive for the respective adverse effects. In fulfilling these prerequisites, the contents of the data base MetaMap^®^Tox, and particularly the specific toxicity patterns, can serve to define an adverse effect based on metabolome data.

### Statistical analysis and interpretation of findings

All quantifiable test results were calculated as means and standard deviations of each test group. Additionally, the following statistical analyses were performed:

- Body weight and body weight change: A comparison of each group with the control group using Dunnett’s test (two sided) for the hypothesis of equal means;
- Blood and urine parameters with uni- or bidirectional changes: One or two-sided Wilcoxon test for the hypothesis of equal medians;
- Blood and urine parameters with unidirectional changes: Pair-wise, one-sided Wilcoxon test for the hypothesis of equal medians;
- Organ weight parameters: A pair-wise comparison of each dose group with the control group was performed using the Wilcoxon test for the hypothesis of equal medians.

In all tests, levels of significance of *p* ≤ 0.05 (*) and *p* ≤ 0.01 (**) were recorded.

To relate statistical findings to true adverse biological effects, the following criteria were applied as specified by ECETOC (2002):

- Establish that there is a difference between the test groups and control groups.
- Is the difference an effect of the treatment or is it incidental (i.e., has it arisen by chance)?—A difference is less likely to be treatment-related if, e.g., it is caused by findings in one or more animals, which could be considered outliers, if it is within the range of historical control values, or if it lacks biological plausibility (i.e., it is inconsistent with known MoAs).
- Is the treatment-related effect adverse?—Findings are less likely to be adverse if, e.g., the general function of the test organism or of the affected organ remains unaffected or findings occur in isolation (i.e., changes in other parameters usually associated with the effect of concern are not observed).

## Results

### Test substance characterization

An overview of the primary and secondary physico-chemical properties of the test substances is provided in Table 1, which has been adapted from Wohlleben et al. (2013) and Landsiedel et al. (2014). Further information on the preparation and characterization of the set of nanoGEM test substances is available from Hellack et al. (2012).

As recorded in Table 1, all test substances were well dispersed in water and had average agglomeration numbers (AAN, i.e., the average number of primary particles in the agglomerate) of 1 (or 3, in the case of ZrO_2·_acrylate). Only BaSO_4_ NM-220, which was not provided as suspension, but as powder, had a higher AAN of 11. In PBS + BSA, SiO_2·_PEG and ZrO_2·_TODS prevailed as large agglomerates above 1 μm, and the BSA concentration in these solution was significantly reduced, indicating BSA adsorption onto the test materials. SiO_2·_naked, however, remained stable in PBS + BSA with only minimal agglomeration, but also here BSA adsorption was recorded. When diluted in DMEM + FCS, only SiO_2·_phosphate remained well dispersed (AAN of 1 or 2), whereas all other NMs were moderately (AAN = 28 and 32 for SiO_2·_naked and ZrO_2·_acrylate, respectively) or strongly agglomerated (AAN = 86, 90, and 213 for ZrO_2·_TODS, SiO_2·_amino and SiO_2·_PEG, respectively).

The iso-electric points of the test substances ranged from pH values above 7 for SiO_2·_amino and ZrO_2·_TODS to between 3 and 4.5 for SiO_2·_PEG and BaSO_4_ NM-220 to below 1 for SiO_2·_naked, SiO_2·_phosphate, and ZrO_2·_acrylate.

### Concentration and homogeneity control analysis

*Sub-study A*: Ranging between 9.6 and 10.3 g test substance per 100 g preparation, the results of the concentration control analysis confirmed the correctness of the concentrations of the surface-functionalized SiO_2_ and ZrO_2_ NMs in the PBS + BSA preparations. Likewise, the overall identical concentrations determined in the bottom, middle, and top layers of the suspensions confirmed that the substances were distributed homogeneously in the vehicles (Table 2).

**Table 2:** Concentration control analysis of test substance suspensions (‘as delivered’ and ‘as prepared’)

Sub-study	Test substance	Concentration control analysis					
		Test substance ‘as-produced’		Test substance ‘as prepared’			
		Concentration declaration; supplier (%)	Concentration control (g/100 g)	Sample 1 (g/100 g)	Sample 2 (g/100 g)	Sample 3 (g/100 g)	Mean of samples 1–3 (g/100 g)
A	SiO_2·_PEG	20	20.1	10.1	10.1	10.1	10.1
A	SiO_2·_phosphate	20	20.4	10.3	10.3	10.3	10.3
A	SiO_2·_amino	20	20.1	10.3	10.3	10.3	10.3
A	ZrO_2·_acrylate	35	33.8	9.6	9.6	9.6	9.6
A	ZrO_2·_TODS	45	43.3	9.9	9.9	10.1	10.0
B	SiO_2·_naked	40	39.2	7.9	7.9	11.6	9.1
B	BaSO_4_ NM-220	Powder	92.7	6.6	7.3	5.4	6.5

In the vials, samples 1–3 are taken from the bottom, middle, and top layers of the suspensions

The mass of the test substances only corresponds to the mass of the core nanomaterial, and not to the molecules of the surface functionalization material that was only present in the preparations in negligible concentrations (Hellack et al. 2012)

*Sub-study B*: As revealed by differing concentrations measured in the three samples, SiO_2·_naked was not distributed homogeneously in the test substance preparations. The mean of all 3 samples, however, confirmed the overall 10 wt% suspension, most likely an indication that the remaining particles had sedimented to the bottom of the vials. Also BaSO_4_ was not distributed homogeneously in the test substance preparations, and only contained an average of 6.5 g BaSO_4_ per 100 g (Table 2).

### Findings recorded in the 28-day oral toxicity study

#### Food and water consumption, clinical examination, body weight gain

In *sub-studies A and B*, food and water consumption of all test animals was unaffected by all applied test substances.

*Sub-study A*: Upon clinical examination, no test substance-related adverse effects were observed in any of the test groups. In the males of the SiO_2·_PEG test group, on study day 28, body weight change values were significantly increased by 20 % as compared to the control group (Table 3). Since this finding did not correspond to any other observable effect, it was considered to be incidental and not treatment-related in accordance with the ECETOC (2002) criteria.

**Table 3:** Body weight; all test substances applied daily at 1,000 mg/kg body weight for 28 days; body weight (g), expressed as mean of test group (*N* = 5, except otherwise indicated)

Day	Male animals						Female animals					
	Control group	SiO_2·_PEG	SiO_2·_ phosphate	SiO_2·_amino	ZrO_2·_ acrylate	ZrO_2·_TODS	Control group	SiO_2·_PEG	SiO_2·_amino	SiO_2·_ phosphate	ZrO_2·_ acrylate	ZrO_2·_TODS
*Sub-study A*												
0	160	161	162	159	162	162	128	128	127	127	129	127
7	202	206	206	203	205	205	141	149	144	147	148	145
14	238	249	249	246	247	247	159	170	165	164	168	163
21	268	286	279	282	285	275	176	184	178	183	185	176
28	282	307*	302	300	306	292	182	192	183	190	192	186

Day	Male animals			Female animals		
	Control group	SiO_2·_naked	BaSO_4_	Control group	SiO_2·_naked (*N* = 4)	BaSO_4_
*Sub-study B*						
0	164	167	169	132	130	134
7	204	211	211	149	151	153
14	239	248	252	168	166	174
21	265	275	280	180	178	191
28	286	295	301	192	183	200

* Significant (*p* ≤ 0.5) body weight change relative to the control group by more than 20 %

*Sub-study B*: One female animal of the SiO_2·_naked test group was found dead on study day 5. The animal died because of a gavage error. In none of the male or female animals of sub-study B, significant changes in regard to mean body weights were observed (Table 3).

### Clinical pathology

#### Hematology

For none of the test groups, recorded changes in hematological parameters were assessed as being related to the treatment with the respective nanomaterials.

*Sub-study A* (Tables 4, 5): In the male animals of the SiO_2·_PEG test group, the mean relative reticulocyte (Ret. %) and platelet counts (PLT) were higher than those of the control group (i.e., 2.6 % as compared to 2.1 %; and 975 giga/L as compared to 851 giga/L), and in the female animals of this test group, the Ret. % counts were decreased (i.e., 2.1 % as compared to 2.5 %). In the males of the SiO_2·_phosphate test group, the PLT counts were elevated (935 vs. 851 giga/L), and in the females of this test group, the hemoglobin (HGB) values were decreased (8.2 mmol/L; control animals: 8.5 mmol/L). Since all of these values were within the respective historical control ranges (Tables 4, 5; *cf.* Supplementary Information for details on the historical control data collected in in-house studies), all alterations were assessed as incidental and not treatment-related in accordance with the ECETOC (2002) criteria. No white blood cell parameters were changed in either the male or the female rats of any test group of sub-study A.

**Table 4:** Hematology: Red blood cell and coagulation parameters (determined on day 29), expressed as mean of test group (*N* = 5; except otherwise indicated)

	Historical control range^a^	Control group	SiO_2·_PEG	SiO_2·_phosphate	SiO_2·_amino	ZrO_2·_acrylate	ZrO_2·_TODS
*Sub-study A*							
Male animals							
RBC; tera/L	7.59–8.60	8.28	8.16	8.27	8.27	8.14	8.10
HGB; mmol/L	8.6–9.5	8.9	8.9	8.9	8.9	8.8	8.9
HCT; L/L	0.384–0.432	0.427	0.428	0.431	0.432	0.429	0.424
MCV; fL	48.1–53.3	51.6	52.5	52.0	52.3	52.7	52.3
MCH; fmol	1.06–1.19	1.07	1.08	1.08	1.07	1.08	1.09
MCHC; mmol/L	20.43–23.73	20.75	20.68	20.70	20.57	20.57	20.90
RET; %	1.4–3.1	2.1	2.6*	2.4	2.3	2.3	2.4
PLT; giga/L	791–1,025	851	975**	935*	807	883	909
HQT; sec	33.3–39.6	38.1	37.5	38.7	38.7	38.2	38.2
Female animals							
RBC; tera/L	7.15–8.12	7.46	7.48	7.47	7.67	7.41	7.34
HGB; mmol/L	8.1–9.1	8.5	8.4	8.2*	8.6	8.4	8.3
HCT; L/L	0.358–0.405	0.403	0.402	0.390	0.404	0.396	0.395
MCV; fL	48.5–53.2	54.1	53.7	52.4	52.8	53.5	53.9
MCH; fmol	1.09–1.22	1.14	1.13	1.10	1.12	1.13	1.13
MCHC; mmol/L	20.99–24.34	20.97	20.95	20.96	21.20	21.21	21.03
RET; %	1.6–4.6	2.5	2.1*	2.3	2.1	2.2	2.5
PLT; giga/L	779–1,022	952	927	877	675	884	877
HQT; sec	30.3–36.7	35.3	35.5	35.6	34.8	34.9	35.8

	Historical control range^a^	Control group	SiO_2·_naked^b^	BaSO_4_
*Sub-study B*				
Male animals				
RBC; tera/L	7.59–8.60	7.94	8.27	8.14
HGB; mmol/L	8.6–9.5	8.9	8.8	9.1
HCT; L/L	0.384–0.432	0.432	0.425	0.440
MCV; fL	48.1–53.3	54.5	51.4	54.1
MCH; fmol	1.06–1.19	1.12	1.07	1.11
MCHC; mmol/L	20.43–23.73	20.58	20.81	20.59
RET; %	1.4–3.1	2.2	2.1	2.2
PLT; giga/L	791–1,025	836	792	844
HQT; sec	33.3–39.6	37.1	33.9	38.4
Female animals				
RBC; tera/L	7.15–8.12	7.32	7.89	7.89
HGB; mmol/L	8.1–9.1	8.2	8.7	8.7**
HCT; L/L	0.358–0.405	0.392	0.414	0.415**
MCV; fL	48.5–53.2	53.6	52.4	52.7
MCH; fmol	1.09–1.22	1.13	1.10	1.11
MCHC; mmol/L	20.99–24.34	21.07	21.10	21.03
RET; %	1.6–4.6	2.4	2.0	2.1
PLT; giga/L	779–1,022	745	796	823
HQT; sec	30.3–36.7	34.2	33.8	34.5

*RBC* red blood cells, *HGB* hemoglobin, *HCT* hematocrit, *MCV* mean corpuscular volume, *MCH* mean corpuscular hemoglobin, *MCHC* mean corpuscular hemoglobin concentration, *RET* reticulocytes, *PLT* platelets, *HQT* prothrombin time (Heptatoquick^®^ test)

*Units* giga/L = 10^9^/liter; tera/L = 10^12^/liter; fL = fentoliter; mmol/L = millimole/liter; fmol = fentomole; L/L = liter/liter; sec = seconds

Significance, as compared to the control group: * p ≤ 0.5 ; ** p ≤ 0.1

^a^Historical control range (*N* = 37 for male and female rats, respectively): unpublished in-house data, see Supplementary Information for details

^b^Female animals of test group SiO_2·_naked: *N* = 4

**Table 5:** Hematology: White blood cell parameters (determined on day 29), expressed as mean of test group (*N* = 5; except otherwise indicated)

	Historical control range^a^	Control group	SiO_2·_PEG	SiO_2·_phosphate	SiO_2·_amino	ZrO_2·_acrylate	ZrO_2·_TODS
*Sub-study A*							
Male animals							
WBC; giga/L	4.38–7.90	4.94	4.22	6.20	4.86	4.57	5.52
LUC; giga/L	0.01–0.05	0.02	0.02	0.03	0.02	0.02	0.02
LUC; %	0.3–0.8	0.4	0.4	0.4	0.4	0.5	0.3
Neut.; %	9.4–16.6	12.5	11.3	14.0	10.3	10.6	11.5
Lymph.; %	79.1–87.0	84.5	85.2	82.5	86.5	85.8	84.9
Mono.; %	0.9–2.5	1.2	1.4	1.6	1.5	1.4	1.5
Eos.; %	1.1–2.8	1.1	1.3	1.1	1.0	1.2	1.3
Baso.; %	0.0–0.9	0.3	0.3	0.3	0.3	0.5	0.4
Female animals							
WBC; giga/L	3.06–5.08	2.84	3.17	3.12	3.28	3.26	3.14
LUC; giga/L	0.01–0.03	0.01	0.01	0.01	0.02	0.01	0.01
LUC; %	0.2–0.7	0.3	0.5	0.4	0.5	0.3	0.4
Neut.; %	7.9–19.5	11.4	8.4	11.3	9.3	9.7	11.8
Lymph.; %	76.3–88.2	85.0	88.4	85.4	87.1	86.8	84.4
Mono.; %	1.0–2.3	1.3	1.0	1.4	1.3	1.1	1.1
Eos.; %	1.0–3.0	1.6	1.4	1.2	1.5	1.6	2.0
Baso.; %	0.0–0.8	0.4	0.3	0.3	0.4	0.4	0.3

	Historical control range^a^	Control group	SiO_2·_naked^b^	BaSO_4_
*Sub-study B*				
Male animals				
WBC; giga/L	4.38–7.90	6.30	5.86	5.49
LUC; giga/L	0.01–0.05	0.04	0.05	0.03
LUC; %	0.3–0.8	0.6	1.0	0.5
Neut.; %	9.4–16.6	12.8	16.6	12.3
Lymph.; %	79.1–87.0	83.0	78.7	83.8
Mono.; %	0.9–2.5	1.7	2.4	1.7
Eos.; %	1.1–2.8	1.5	1.0*	1.1
Baso.; %	0.0–0.9	0.4	0.3	0.5
Female animals				
WBC; giga/L	3.06–5.08	3.97	4.99	4.95
LUC; giga/L	0.01–0.03	0.02	0.04	0.04*
LUC; %	0.2–0.7	0.5	0.8	0.8*
Neut.; %	7.9–19.5	19.1	24.1	10.5
Lymph.; %	76.3–88.2	77.5	71.1	84.7
Mono.; %	1.0–2.3	1.2	2.0	1.5
Eos.; %	1.0–3.0	1.3	1.8	2.1
Baso.; %	0.0–0.8	0.4	0.3	0.5

*WBC* white blood cells, *LUC* large unstained cells, *Neut.* polymorphonuclear neutrophils, *Lymph.* lymphocytes, *Mono.* monocytes, *Eos.* eosinophils, *Baso.* basophils

*Units*: giga/L = 10^9^/liter

Significance, as compared to the control group: * *p* ≤ 0.5

^a^Historical control range (*N* = 37 for male and female rats, respectively): unpublished in-house data, see Supplementary Information for details

^b^Female animals of test group SiO_2·_naked: *N* = 4

*Sub-study B* (Tables 4, 5): No red blood cell or coagulation parameters were changed in the male rats of sub-study B. Regarding white blood cell parameters, in the male animals of the SiO_2·_naked test group, relative eosinophil (Eos.%) counts were lower as compared to the control group (1.0 vs. 1.5 %; Tables 5). The recorded value, however, was only marginally below the historical control range (1.1–2.8 %), the absolute eosinophil counts were not altered (data not shown), and none of the other differential blood cell fractions were changed. Furthermore, the eosinophil counts were not changed in the female animals of the same test group. Therefore, these alterations were regarded as incidental and not treatment-related in accordance with the ECETOC (2002) criteria.

In the female animals of the BaSO_4_ test group, the HGB and hematocrit (HCT) values (8.7 mmol/L and 0.415 L/L) as well as the absolute and relative large unstained cell (LUC) counts (0.04 giga/L and 0.8 %) were higher than those recorded for the related control group (HGB: 8.2 mmol/L; HCT: 0.392 L/L; absolute and relative LUC: 0.02 giga/L and 0.5 %; Tables 4, 5). Whereas the HGB and HCT values were within historical control ranges (8.1–9.1 mmol/L and 0.358–0.405 L/L, respectively), the LUC counts were slightly higher than the historical control values (absolute and relative LUC: 0.01–0.03 giga/L and 0.2–0.7 %). Since the LUC were the only fraction of the differential blood cell count that was altered and the LUC counts in the male rats of the BaSO_4_ test group were unchanged, all of these hematological alterations were also assessed as incidental and not treatment-related in accordance with the ECETOC (2002) criteria.

#### Clinical chemistry and acute phase proteins

For none of the test groups, recorded changes in clinical chemistry parameters or the acute phase proteins were assessed as being adverse and related to the NM treatment.

*Sub-study A* (Table 6): In the male animals of the SiO_2·_PEG and ZrO_2·_TODS test groups, the chloride levels were lower than those recorded for the control group (102.7 and 102.9 mmol/L, vs 104.9 mmol/L). In the female animals of the ZrO_2·_acrylate test group, alanine aminotransferase (ALT) activities were increased (0.68 µkat/L as compared to 0.53 µkat/L), and in the female animals of the SiO_2·_phosphate test group, total protein, albumin, and globulin levels were decreased (59.27, 39.68, and 19.59 g/L, respectively, as compared to 64.91, 43.02, and 21.90 g/L). Apart from the globulin levels recorded for the female animals of the SiO_2·_phosphate group, all of these findings were within the respective historical control ranges (male rats: chloride: 99.2–104.0 mmol/L; female rats: ALT: 0.46–0.80 µkat/L; total protein: 58.40–66.16 g/L; albumin: 35.44–40.66 g/L; globulin: 21.07–26.27 g/L; Table 6 and Supplementary Information). Therefore, they were regarded as incidental and not treatment-related. Since the globulin levels were in fact marginally below the historical control range, this alteration was regarded as possibly treatment-related, but not adverse (ECETOC 2002).

**Table 6:** Clinical chemistry (i.e., enzymes, electrolytes and minerals, substrates, acute phase proteins; determined on day 29), expressed as mean of test group (*N* = 5; except otherwise indicated)

	Historical control range^a^	Control group	SiO_2·_PEG	SiO_2·_phosphate	SiO_2·_amino	ZrO_2·_acrylate	ZrO_2·_TODS
*Sub-study A*							
Male animals							
ALT; µkat/L	0.53–0.89	0.62	0.63	0.65	0.65	0.63	0.73
AST; µkat/L	1.46–2.42	1.82	1.70	1.89	1.81	1.66	1.85
AP; µkat/L	1.50–2.80	2.79	2.37	2.72	2.46	2.58	2.17
gGT; nkat/L	0–13	0	0	0	0	0	0
TP; g/L	59.09–65.07	62.25	61.53	62.63	60.85	62.10	61.74
Alb; g/L	35.28–38.41	40.27	39.57	40.14	39.63	40.09	39.73
Glob; g/L	22.15–28.70	21.98	21.96	22.50	21.22	22.01	22.01
Na; mmol/L	139.1–146.0	146.4	145.2	145.7	145.1	146.1	144.6
K; mmol/L	4.29–4.91	4.38	4.61	4.37	4.56	4.41	4.52
Cl; mmol/L	99.2–104.0	104.9	102.7*	103.2	103.6	103.2	102.9*
INP; mmol/L	1.88–2.39	1.98	2.05	2.07	1.99	1.94	2.04
Ca; mmol/L	2.42–2.70	2.52	2.51	2.53	2.46	2.50	2.49
Urea, mmol/L	4.69–7.67	5.90	5.54	5.81	5.49	5.43	5.51
Crea; µmol/l	43.9–52.5	49.0	47.3	49.0	47.5	48.1	50.1
TBIL; µmol/L	1.46–2.63	2.29	2.96	2.66	2.23	2.75	2.52
Hapt; ng/mL	265.2–1,074.0	1,523.0	853.0	1,399.8	1,383.80	1,837.6	1,724.0
α2 m; ng/mL	8.92–33.77	15.68	14.91	15.17	14.79	13.86	15.59
Female animals							
ALT; µkat/L	0.46–0.80	0.53	0.53	0.51	0.69	0.68*	0.67
AST; µkat/L	1.36–2.69	1.85	1.70	1.88	2.34	2.21	2.09
AP; µkat/L	0.71–2.01	1.56	1.56	1.65	1.46	1.59	1.62
gGT; nkat/L	0–17	1	0	1	0	0	0
TP; g/L	58.40–66.16	64.91	63.87	59.27**	62.06	62.76	62.61
Alb; g/L	35.44–40.66	43.02	42.60	39.68**	41.26	41.53	41.91
Glob; g/L	21.07–26.27	21.90	21.27	19.59*	20.80	21.22	20.70
Na; mmol/L	138.9–144.3	145.9	146.0	144.9	145.3	146.2	146.5
K; mmol/L	3.79–4.41	4.11	4.16	4.03	4.16	3.98	4.16
Cl; mmol/L	100.1–104.8	106.0	105.9	106.0	105.1	105.9	106.5
INP; mmol/L	1.52–2.01	1.78	1.84	1.89	1.82	1.69	1.80
Ca; mmol/L	2.43–2.65	2.50	2.55	2.45	2.53	2.59	2.49
Urea, mmol/L	5.74–8.49	5.65	5.89	5.67	5.58	6.07	5.55
Crea; µmol/l	47.0–56.1	49.2	47.7	51.3	48.7	51.6	49.1
TBIL; µmol/L	1.51–2.88	1.26	0.96	0.83	1.43	1.21	1.39
Hapt; ng/mL	Not available	695.0	403.6	585.8	453.0	698.2	689.0
α2 m; ng/mL	Not available	16.93	15.78	12.46	17.84	14.72	16.32

	Historical control range^a^	Control group	SiO_2·_naked^b^	BaSO_4_
*Sub-study B*				
Male animals				
ALT; µkat/L	0.53–0.89	0.80	0.76	0.80
AST; µkat/L	1.46–2.42	1.99	2.10	2.26
AP; µkat/L	1.50–2.80	2.35	1.99	2.39
gGT; nkat/L	0–13	0	0	0
TP; g/L	59.09–65.07	60.20	62.34	62.76
Alb; g/L	35.28–38.41	37.53	38.20	39.02
Glob; g/L	22.15–28.70	22.67	24.13	23.74
Na; mmol/L	139.1–146.0	141.8	140.5	141.8
K; mmol/L	4.29–4.91	4.45	4.52	4.59
Cl; mmol/L	99.2–104.0	101.7	100.4	100.9
INP; mmol/L	1.88–2.39	2.15	2.01	2.15
Ca; mmol/L	2.42–2.70	2.53	2.53	2.54
Urea, mmol/L	4.69–7.67	5.77	6.23	5.54
Crea; µmol/l	43.9–52.5	45.1	47.5	46.5
TBIL; µmol/L	1.46–2.63	1.40	1.38	1.28
Hapt.; ng/mL	265.2–1,074.0	234.0	413.6*	374.3*
α2 m; ng/mL	8.92–33.77	11.08	14.29	11.25
Female animals				
ALT; µkat/L	0.46–0.80	0.59	0.54	0.52
AST; µkat/L	1.36–2.69	1.95	1.87	1.71
AP; µkat/L	0.71–2.01	1.19	0.91	0.93
gGT; nkat/L	0–17	0	0	0
TP; g/L	58.40–66.16	61.05	61.07	62.08
Alb; g/L	35.44–40.66	39.44	39.17	40.23
Glob; g/L	21.07–26.27	21.61	21.90	21.85
Na; mmol/L	138.9–144.3	140.0	139.0	139.9
K; mmol/L	3.79–4.41	4.01	3.92	4.09
Cl; mmol/L	100.1–104.8	101.9	99.6	100.8
INP; mmol/L	1.52–2.01	1.68	1.68	1.83
Ca; mmol/L	2.43–2.65	2.50	2.52	2.56
Urea, mmol/L	5.74–8.49	6.06	5.66	6.35
Crea; µmol/l	47.0–56.1	51.6	50.7	49.2
TBIL; µmol/L	1.51–2.88	1.08	0.97	1.60
Hapt.; ng/mL	Not available	141.7	469.5	153.3
α2 m; ng/mL	Not available	12.65	20.80	13.89

*ALT* alanine aminotransferase, *AST* aspartate aminotransferase, *AP* alkaline phosphatase, *gGT* gamma glutamyl transferase; *TP* total protein, *Alb.* albumin, *Glob.* globulins, *Na* sodium, *K* potassium, *Cl* chloride, *INP* inorganic phosphate, *Ca* calcium, *Crea.* creatinine, *TBIL* total bilirubin, *Hapt.* haptoglobin, *α2m* α2-macroglobin

Significance, as compared to the control group: * *p* ≤ 0.5 ; ** *p* ≤ 0.1

*Units:* µkat/L = microkatal per liter; nkat/L = nanokatal per liter; ng/mL = nanogram per milliliter; g/L = gram per liter; mmol/L = millimole per liter

^a^Historical control range (*N* = 37 for male and female rats, respectively, except for acute phase proteins: *N* = 23 for male rats, and no historical controls available for female rats): unpublished in-house data, see Supplementary Information for details

^b^Female animals of test group SiO_2·_naked: *N* = 4

In none of the test groups of sub-study A, significant changes in the haptoglobin, α2-macroglobin, or troponin I levels were recorded. For cardiac troponin I, all values were furthermore below the sensitivity level of the assay. Therefore, this parameter was excluded from the evaluation in sub-study B (and the parameter is not recorded in Table 6).

*Sub-study B* (Table 6): In the male animals of the SiO_2·_naked and BaSO_4_ test groups, haptoglobin values were higher than those recorded for the control group (413.6 and 374.3 ng/mL, respectively, as compared to 234.0 ng/mL). However, these haptoglobin values were within the historical control range (haptoglobin: 265.2–1.074.0 ng/mL, corresponding to a range of fourfold between the lowest and the highest mean of control groups previously recorded in unpublished in-house studies; *cf.* Supplementary Information). Of note, the mean haptoglobin value recorded for the control group of sub-study A (1,523.0 ng/mL) exceeded this historical control range value, whereas the mean haptoglobin value recorded for the control group of sub-study B (234.0 ng/mL) was slightly below it. In regard to the present survey, these observations were assessed as incidental in accordance with the ECETOC (2002) criteria, and they might be an indication that the historical control range should be considered as preliminary.

In the female rats of the SiO_2·_naked test group, the mean haptoglobin values were also considerably higher than those recorded for the respective control group (469.5 ng/mL as compared to 141.7 ng/mL). Assessment of the implications of these values is impaired by the circumstance that historical control ranges for haptoglobin values in female rats were neither available in-house, nor could be found in the published literature. Additionally, one of these animals not only had an extremely high haptoglobin value (i.e., 1,610.2 ng/mL; data for individual animals not shown), but also a high α2-macroglobulin value (49.21 ng/mL as compared to the mean control value of 12.65 ng/mL) and increased total white blood cell (WBC) and (absolute and relative) neutrophil counts [WBC: 8.44 as compared to 3.97 giga/L; neutrophils: 5.36 giga/L (63.5 %) as compared to 0.66 giga/L (19.1 %)]. Upon necropsy, no macroscopic or histopathological correlates to these findings could be determined. Therefore, this individual animal was diagnosed as most likely having had a systemic inflammation. In addition, for the other three (remaining) animals of this test group (one animal of this test group had died due to gavage error), all mentioned parameters were within the normal ranges, and there were no histopathologically relevant findings upon necropsy. (When excluding the haptoglobin value of 1,610.2 ng/mL, the mean haptoglobin value of the remaining animals of the SiO_2·_naked test group amounted to 89.3 ng/mL, i.e., it was even lower than the mean control value). Therefore, the mean haptoglobin value of the female rats of the SiO_2·_naked test group, just as the altered parameters of the individual female rat, was assessed as not being related to the test substance SiO_2·_naked in accordance with the ECETOC (2002) criteria.

### Urinalysis

Neither in *sub-studies A nor B*, any treatment-related, adverse changes of any of the urine parameters assessed were recorded.

### Pathology

#### Absolute and relative organ weights

*Sub-study A*: When compared to the respective control groups (whose organ weights were set as 100 %), the absolute weight of the epididymides was significantly decreased in the male rats of the SiO_2·_PEG test group, and the absolute weights of the heart and spleen were increased in the male rats of the SiO_2·_amino test group (Table 7). Also in regard to relative organ weight, the weight of the epididymides of the SiO_2·_PEG test group was decreased. Furthermore, the relative weights of the prostate and testes of the male animals of the ZrO_2·_acrylate test group were decreased (Table 8).

**Table 7:** Pathology (absolute weights, expressed as mean of test group (*N* = 5) relative to respective control group)

Affected organ	Control group (%)	SiO_2·_PEG (%)	SiO_2·_phosphate (%)	SiO_2·_amino (%)	ZrO_2·_acrylate (%)	ZrO_2·_TODS (%)
*Sub-study A*						
Male animals						
Epididymis	0.706 g = 100	90*	100	106	96	101
Heart	0.802 g = 100	113	102	111*	108	108
Spleen	0.454 g = 100	111	119	124*	114	112
Female animals						
Uterus	0.746 g = 100	74	62*	75	74	74

For sub-study B, there are no significant increases or decreases of the mean absolute organ weights in any of the test groups as compared to the respective control group values

For all other organs (i.e., adrenal glands, brain, kidneys, liver, ovaries (female), prostate (male), seminal vesicle (male), testes (male), thymus, thyroid glands) and the terminal body weight, no significant changes in absolute weights, relative to the control group, are determined

Significance, as compared to the control group: * *p* ≤ 0.5

**Table 8:** Pathology (relative organ weights; expressed as mean of test group (*N* = 5; except otherwise noted) and relative to respective control group)

Control group; terminal body weight (g)	Control group; organ weight relative to terminal body weight (%)	Affected organ	SiO_2·_PEG (%)	SiO_2·_phosphate (%)	SiO_2·_amino (%)	ZrO_2·_acrylate (%)	ZrO_2·_TODS (%)
*Sub-study A*							
Male animals							
256 g = 100 %	0.27	Epididymis	82**	93	99	88	96
	0.23	Prostate	90	88	90	73*	100
	1.19	Testes	89	94	95	90*	99
Female animals							
168 g = 100 %	0.82	Kidneys	94	92*	92**	90**	94
	0.23	Thymus	112	93	83*	104	113

Control group; terminal body weight (g)	Control group; organ weight relative to terminal body weight	Affected organ	SiO_2·_naked	BaSO_4_
*Sub-study B*				
Male animals				
264 g = 100 %	2.45	Liver	111*	108

Control group; terminal body weight (g)	Control group; organ weight relative to terminal body weight	Affected organ	SiO_2·_naked (*N* = 4)	BaSO_4_
Female animals				
176 g = 100 %	0.36	Heart	100	94*

Relative organ weight: organ weight relative to terminal body weight of the individual animal

Significance, as compared to the control group: * p ≤ 0.5; ** p ≤ 0.1

For the female rats, the mean absolute weight of the uterus was decreased in the SiO_2·_phosphate test group (assessed as being cycle-related), and the relative weights of the kidneys and thymus were decreased in the animals of the SiO_2·_amino and Zr_·_O_2·_acrylate (only kidney) test groups (Tables 7, 8).

All of these changes were assessed as not being treatment-related in accordance with the ECETOC (2002) criteria since they did not correspond to any histopathological findings and since (apart from the reduced weight of the epididymides of the SiO_2·_PEG test group) absolute and relative changes in organ weight did not correlate.

*Sub-study B*: There were no significant increases or decreases of the mean absolute organ weights in any of the test groups as compared to the respective control group values. In regard to mean relative organ weights, the weight of the liver of the male animals of the SiO_2·_naked test group was increased and the hearts of the female animals of the BaSO_4_ test group were decreased (Table 8). Again, all of these changes were assessed as not being treatment-related in accordance with the ECETOC (2002) criteria since there were no corresponding changes in absolute organ weights or histopathological findings that would explain the weight changes.

#### Gross lesions

For *sub-study A*, all gross lesions were single observations, and they were regarded to have developed spontaneously and to be unrelated to either the test compounds or the administration procedure.

*Sub-study B*: One female animal of the SiO_2·_naked test group died ahead of schedule having a thoracic effusion that was assessed as having been caused by a gavage error. Therefore, this finding was regarded as related to the administration procedure, but not to the test substances. All other gross lesions noted were single observations, and they were regarded to have developed spontaneously and to be unrelated to the test substances or the treatments (data not shown).

#### Histopathology

*Sub-study A* (Table 9): All findings noted were either single observations or they were equally distributed between control and treatment groups. All of the findings were considered to be incidental or spontaneous in origin and without any relation to the test substance treatment.

**Table 9:** Histopathology (incidence of microscopic findings)

	Male animals						Female animals					
	Control group	SiO_2·_PEG	SiO_2·_phosphate	SiO_2·_amino	ZrO_2·_acrylate	ZrO_2·_TODS	Control group	SiO_2·_PEG	SiO_2·_phosphate	SiO_2·_amino	ZrO_2·_acrylate	ZrO_2·_TODS
*Sub-study A*												
No. of animals	5	5	5	5	5	5	5	5	5	5	5	5
Glandular stomach Metaplasia, basal cells				1								
Dilation of glands	1	1			1		2			1		
Kidneys Infiltration lymphoid		1			3					1		
Cast, tubular					1							
Scar(s), cortical											1	
Cysts					1							
Liver Necrosis					1							
Peri-/vasculitis		1			1	1		1				
Fatty change, (multi)focal		1										
Cecum Parasites in lumen		1	1									
Prostate Infiltr., lymphoid	1				1							
Seminal vesicle Content reduced					2a							

	Male animals			Female animals		
	Control	SiO_2·_naked	BaSO_4_	Control	SiO_2·_naked	BaSO_4_
*Sub-study B*						
No of animals	5	5	5	5	5	5
Glandular stomach Discoloration of contents						
Inflammatory. cell infiltrates	3	2	3			4
Edema						1
Dilation of glands				2		
Infiltration, lymphoid	2					1
Kidneys Cast, tubular	1					
Cyst(s), cortical		1		1	1	
Dilation, tubular	1		1	1	1	
Dilation, renal pelvis			1			
Liver Necrosis, (multi)focal	1					
Peri-/vasculitis	1	1				
Fatty change, (multi)focal						
Duodenum Particles submucosa/macrophages						
Jejunum Discoloration of contents						
Ileum Discoloration of contents						
Cecum Discoloration of contents						
Rectum Discoloration of contents						

a: Organs from 2 animals investigated

*Sub-study B* (Table 9): In the female animals of the BaSO_4_ test group, minimal to slight inflammatory cell infiltrates in the submucosa of the glandular stomach were observed. All other findings were either single observations or they were equally distributed between the control and treatment groups. Therefore, all observations were considered to be incidental or spontaneous in origin and unrelated to the treatment.

### Metabolome analysis

At a dose level of 1,000 mg/kg, none of the tested NMs had a biologically relevant impact on the plasma metabolome pattern of rats. When compared against the control animals on a significance level of *p* < 0.05, in both male and female animals, the number of significantly changed endogenous metabolites was below or at the false positive rate for all particles tested and were assessed as “statistical variance” of the metabolome analysis (Table 10). Using the pattern ranking, i.e., matching the metabolome of the compounds with pre-defined patterns of metabolite changes, which are associated with adverse effects, there were no common, consistent matches with any pattern for any of the investigated substances (data not shown).

**Table 10:** Numbers of significantly changed endogenous metabolites in rat plasma (*p* < 0.05)

Test substance (1,000 mg/kg bw/day)	Sex	Numbers of up-regulated endogenous metabolites	Numbers of down-regulated endogenous metabolites
SiO_2·_PEG	Male	7	1
	Female	4	7
SiO_2·_phosphate	Male	7	0
	Female	4	1
SiO_2·_amino	Male	4	1
	Female	11	5
ZrO_2·_acrylate	Male	3	0
	Female	3	1
ZrO_2·_TODS	Male	7	1
	Female	2	2
SiO_2·_naked	Male	2	11
	Female	3	20
BaSO_4_	Male	4	1
	Female	1	4

## Discussion

Test substance-related adverse effects were determined for none of the seven nanomaterials applied in the 28-day oral rat toxicity study, i.e., 4 SiO_2·_NMs (the core material and 3 variants with surface functionalization), 2 surface-functionalized ZrO_2_ NMs and BaSO_4_ NM-220, neither in assessing the standard parameters listed in OECD TG 407 nor by metabolic profiling as a means of detecting changes at very early stages of their evolvement (*cf.* overview in Table 11).

**Table 11:** Overview of findings per test groups upon 28-day oral gavage administration of 1,000 mg/kg/day of the respective nanomaterials assessed as incidental or test substance-related (see Tables 3, 4, 5, 6, 7, 8, 9 for details on the respective findings)

	SiO_2·_naked		SiO_2·_PEG		SiO_2·_ phosphate		SiO_2·_amino		ZrO_2·_acryate		ZrO_2·_TODS		BaSO_4_	
	M	F	M	F	M	F	M	F	M	F	M	F	M	F
Body weight			**b.w. change day 28**											
RBC para-meters			**Ret** **% PLT**	*Ret* *%*	**PLT**	*HGB*								**HGB HCT**
WBC para-meters	*Eos* *%*													**rel./abs. LUC**
Clinical chemistry	**Hapt.**	[**Hapt.**]	*Chloride*			*Globulin,* *TP, albumin*				**ALT**	*Chloride*		**Hapt.**	
Urine analysis								*pH, volume*						
								**spec. grav.**						
Abs. organ weights	**Liver**		*Epididymis*			*Uterus*	**Heart, spleen**							
Rel. organ weights			*Epididymis*					*Kidneys Thymus*	*Prostate Testes*	*Kidneys*				*Heart*
Gross lesions					Enlarged epididymis	Ulcer glandular stomach	Foci in epididymis		Liver torsion, prostate: discolored and size reduced					
Histopathology			(Kidney, liver)	(Liver)			(Kidney, liver, prostate, seminal vesicle)			(Kidney)	(Liver)			

Bold: values increased relative to respective control group (*cf* Tables 3, 4, 5, 6, 7, 8)

Italic values decreased relative to respective control group (*cf.* Tables 3, 4, 5, 6, 7, 8)

Underlined: finding that was assessed as treatment-related (but not adverse)

Gross lesions and histopathology: indication of findings or changed organs (*cf.* Table 9)

*b.w.* body weight, *Ret* reticulocytes, *PLT* platelets, *HGB* hemoglobin, *HCT* hematocrit, *Eos* eosinophilic cells, *f* female, *LUC* large unstained cells, *m* male, *Hapt* haptoglobin, *TP* total protein, *ALT* alanine aminotransferase, *spec. grav.* specific gravity

In interpreting the relevance of these observations for human safety assessment, a number of issues have to be taken into account: (1) Do the physico-chemical characteristics of the nanomaterials in the test substance preparations reflect those of the same NMs as they would enter the human body? Are nanomaterial test substance preparation and characterization sufficiently standardized to ensure reproducibility of test results in accordance with specified parameters? (2) How high is the extent of systemic uptake of the tested nanomaterials and the resulting likelihood to cause adverse effects outside the gastrointestinal tract?

In the following, these issues are addressed in further detail supplemented by a discussion of the results from repeated-dose oral toxicity studies and metabolome studies investigating NM effects from other research groups.

### Nanomaterial preparation and characterization for oral studies

It was an important scope of the overall nanoGEM project, as a part of which the present study was conducted, to select surface-functionalized test substances prevailing in non-agglomerated conditions in their as-produced state. Besides aiming to recognize structure–activity relationships, this served to ensure test result comparability while taking into account that dispersion of the NMs in different test media would affect their colloidal properties, i.e., their state of agglomeration (Wohlleben et al. 2014).

For the oral route of uptake, different NM exposure scenarios are foreseeable. As additives to food or feed, NMs might (or might not) be transformed (e.g., by dissolution) already before ingestion or during digestion (EFSA 2011). It has been cautioned that appropriate data for risk assessment of NMs in the food and feed area should include a comprehensive identification and characterization of the NMs, information on whether they are likely to be ingested in nanoform, and, if absorbed, whether they will prevail in nanoform during absorption (EFSA 2009). The European Food Safety Authority has recommended that NMs in the food and feed area should ideally be characterized in five stages, i.e., as manufactured, as delivered for use in food or feed products, as present in the food and feed matrix, as used in toxicity testing, and as present in biological fluids and tissues (EFSA 2011).

To date, the specific influences of the various physico-chemical conditions met during NM passage through the gastrointestinal tract on their biokinetic properties are largely unknown making it difficult to predict if and how different NMs will be adsorbed (Savolainen et al. 2013). Nanoparticle size, surface properties, including charge, and dissolution all have been recognized as factors that affect NM uptake into the body upon ingestion. NMs reaching the gastrointestinal tract are mostly excreted with the feces, but for some NMs, low levels of absorption have been observed. When such particles become systemically available, they might potentially induce systemic effects (Landsiedel et al. 2012b).

Determination of *nanoparticle agglomeration* plays a major role in assessing the fate of NMs upon oral uptake: Since NM adsorption in the gastrointestinal tract decreases with increasing particle size, NM agglomeration influences bioavailability of the original particles. The rate of NM agglomeration in different vehicles is affected by the pH value of the respective environment (Landsiedel et al. 2012b; Wohlleben et al. 2013).

In the gastrointestinal tract, the intraluminal pH changes rapidly from being highly acidic in the stomach to about pH 6 in the duodenum from where it gradually increases to about pH 7.4 in the terminal ileum (Fallingborg 1999). As regards culture media, PBS + BSA has a pH value of 7.4; DMEM + FCS also has a pH value of 7.4, and it rises to 8 after 24 h (supplier information and in-house recordings). All test substances of the present study, except for SiO_2·_phosphate, agglomerated in DMEM + FCS. In PBS + BSA, especially the NMs of low net charge (SiO_2·_PEG and ZrO_2·_TODS) formed large agglomerates, whereas SiO_2·_naked remained well dispersed. Since the pH of either culture medium resembles the environment of the lower small intestine, it is unlikely that the more strongly agglomerated NMs undergo significant adsorption in the small intestine.

Coco et al. (2013) observed polymer coatings to reduce pH sensitivity of ovalbumin NMs. PEG-ylated NMs were less subject to aggregation due to steric repulsion, and PEG chains were found toward the inner surface of the aqueous phase containing the albumin. Hence, the PEG barrier was able to protect the protein from the denaturing effect of organic solvents.

Wohlleben et al. (2013) have found strongly negative zeta potentials of NMs, confirmed by iso-electric potentials below pH 4, to correlate with near-perfect dispersion in water. At the same time, however, materials with these characteristics (e.g., BaSO_4_ NM-220) were observed to agglomerate rather more than less in the presence of serum (i.e., DMEM + FCS). By contrast, acrylic acid-copolymer-coated SiO_2_ was perfectly dispersed in either water or culture medium since the polymer corona prevented the spontaneous adsorption of a protein corona (Wohlleben et al. 2013). Both homogeneous agglomeration (particle–particle) and heterogeneous agglomeration (particle with dissolved organics) contribute to increased diameters. Consequently, not only NMs with vanishing charge-stabilization due to an iso-electric potential close to the pH of the surrounding media, but also positively charged NMs with strong corona adsorption can have increased diameters.

The pH value of the medium also affects *particle dissolution*, which is a further important parameter affecting NM uptake into the body. Ion release has been recognized as correlating with increased NM mobility in the body and inflammation potency (Wohlleben et al. 2013). In the acidic environment of the stomach, BaSO_4_ particles dissolve, whereas SiO_2_ will partially dissolve already in a neutral environment (Wohlleben et al. 2013).

Evidently, also homogeneity of the test substance preparations influences NM uptake upon oral delivery. Oral NM application by gavage (instead of administration in the food or drinking water) has been recommended as being suitable to ensure well-defined conditions of the test substance administration, since it allows delivering a fairly precise dose of the NM to the animals by a well-characterized degree of dispersion (EFSA 2011). Nevertheless, it has been cautioned that NM application by gavage does not reflect the lower test substance concentrations delivered over longer periods of time when NMs are administered with the animals’ feed: Gavage application provides a bolus of NMs at a given time point that may or may not mix with the gastrointestinal fluids, thereby possibly resulting in a higher local NM concentration and hence increased quantity of the absorbed material (EFSA 2011).

Whereas homogeneity of the test substance preparations was satisfactory in the surface-functionalized SiO_2_ and ZrO_2_ NMs, the non-surface-functionalized SiO_2·_naked and the powder BaSO_4_ were not distributed homogeneously. Suitable standardized protocols remain to be determined to ensure homogenous distribution of NMs with different surface properties in test substance preparations for oral application and to enable determining in which form a given NM will reach the different parts of the gastrointestinal tract.

### Nanomaterial effects upon repeated oral exposure

Since test substance organ burden was not determined in the present study, it is not possible to distinguish whether the lack of adverse effects for any of the tested NMs is an indication that the test substances did not elicit systemic toxicity even though they were present in relevant target organs, or whether they were not taken up into the body to a relevant proportion. Comparisons of the outcome of the present study to other published observations are hardly possible: As regards nanoforms of ZrO_2_ or BaSO_4_, no information on oral repeated-dose toxicity studies was found in the published literature. However, also by the inhalational route of exposure, in a rat short-term inhalation study (5-day inhalation exposure for 6 h/day with a 3-week post exposure period) on the identical materials as in the present study, neither ZrO_2·_TODS, nor ZrO_2·_acrylate, or BaSO_4_ induced any treatment-related effects up to an aerosol concentration of 50 mg/m^3^ (Landsiedel et al. 2014). Due to its reported lack of toxicity and on account of its extensive characterization in the OECD sponsorship programme, BaSO_4_ was used as ‘negative control’ reference material in the present study.

As regards SiO_2_ NMs, the observation that neither the naked core material nor its surface-functionalized variants induced treatment-related adverse effects upon repeated gavage administration is supported by a 28-day oral toxicity study conducted by van der Zande et al. In this study, rats were exposed to 2,500 mg/kg bw/day^1^ of a commercially available amorphous SiO_2_ NMs (PPS: 7 nm; in water, ≥78 % of the material <100 nm (SEM), in feed matrix, approximately 40 % was 5–200 nm (ICP–MS)) or to 1,000 mg/kg SiO_2_ NM-202 (PPS: 10–25 nm; in water, ≥61 % <100 nm (SEM), in the feed matrix, approximately 100 % between 5 and 200 nm). After 28-day oral administration, these test substance concentrations did not result in distinctly elevated SiO_2_ levels in the body tissues, and there were no observable adverse effects. After 84 days of exposure, however, SiO_2_ accumulated in the spleen of the rats treated with the commercially available amorphous SiO_2_, and a significant increase in the occurrence of liver fibrosis was observed in those rats treated with SiO_2_ NM-202 (van der Zande et al. 2014).

By comparison, upon inhalation in a rat STIS 50 mg/m^3^ SiO_2·_naked caused slight and transient increases in granulocyte counts in the blood and increased polymorphonuclear neutrophils and lymphocyte counts in the bronchoalveolar fluid (BALF) shortly after exposure and 3 weeks post exposure, whereas there were no discernible effects at lower aerosol concentrations. Histologically, multifocal macrophage aggregates were observed in the lung shortly after exposure that exacerbated toward a slight multifocal inflammation after the 3-week exposure-free period. At the same dosage, the surface-functionalized SiO_2·_PEG, SiO_2·_phosphate, and SiO_2·_amino did not induce adverse effects in the STIS (Landsiedel et al. 2014).

To date, only few reports on in vivo effects of NMs upon subacute or sub-chronic oral exposure are available, and hardly any investigations addressed the reversibility of effects. The majority of these investigations address the effects of silver nanoparticles, some of which also investigated Ag organ burden. Ag NMs have gained commercial importance, e.g., in food packaging products, because of their antimicrobial activities (Anon 2012; van der Zande et al. 2012). In assessing the hazard of silver NMs, the extent of particle dissolution has been suggested to be the decisive factor determining oral Ag uptake and the severity of resulting systemic effects (Hadrup et al. 2012a; Hadrup and Lam 2014).

Van der Zande et al. (2012) exposed rats orally for 28 days to 90 mg/kg Ag NMs that were either uncoated (<20 nm, TEM; 59 nm, DLS) or coated with polyvinylpyrrolidone (PVP; <15 nm, TEM; 49 nm, DLS). Immediately after the dosing period of either test substance, silver was present in all examined organs. The highest levels of NMs were recorded in the liver and spleen, but without corresponding signs of hepatotoxicity or immunotoxicity. Silver concentrations in the organs highly correlated with the amount of Ag^+^ in the Ag NM suspension, indicating that mainly silver ions passed the intestines. Eight weeks after dosing, silver was cleared from most organs, but not from the brain or testis (van der Zande et al. 2012).

Similarly, 9 mg/kg of PVP-coated Ag NMs (14 nm, DLS) did not induce any toxicological effects upon 28-day oral administration to rats (Hadrup et al. 2012a). For Ag acetate, however, (reflecting ionic silver), lower body weight gain, increased plasma alkaline phosphatase (AP), decreased plasma urea, and lower absolute and relative thymus weights were recorded (Hadrup et al. 2012a). Silver granules containing selenium and sulfur were found in the intestinal walls of the rats exposed to either substance (Loeschner et al. 2011).

In a combined oral repeated-dose—reproduction/developmental toxicity study performed in accordance with OECD TG 422 that was conducted for 42 days in male rats and for 52 days in female rats, up to 250 mg/kg citrate-capped Ag NMs (8 nm, TEM; size distribution in deionized water 5–50 nm, submicron particle size analyzer) did not affect any of the hematological or clinical biochemistry parameters investigated and neither induced histopathological changes nor clinically observable effects (Hong et al. 2014).

Kim et al. (2008) observed changes in AP and cholesterol at concentrations exceeding 300 mg/kg Ag NMs (60 nm; method of particle characterization not indicated) when administered orally to rats for 28 days, as well as an increase in silver in all tissues examined (with accumulation in the kidneys being twofold higher in females than in males). Similar effects were observed upon 90-day oral exposure of rats to 30–500 mg/kg Ag NMs (56 nm, TEM; Kim et al. 2010). These studies by Kim et al., however, have been criticized for insufficient particle characterization impairing the value of the test results (Hadrup et al. 2012a; Dekkers et al. 2013).

In mice exposed orally for 14 days to up to 1.0 mg/kg of different Ag NMs (22, 42, 71, and 323 nm, DLS), the smaller-sized NMs were distributed to brain, lung, liver, kidney, and testis, whereas 323 nm Ag was not. Oral exposure of mice to the same Ag NMs for 28 days (also up to 1.0 mg/kg) affected the livers and kidneys (determined by changes in clinical chemistry parameters and histopathology) and increased cytokine levels (Park et al. 2010). Also in an in vitro co-culture model of the intestinal epithelium, silver ions were found to be the predominant fraction of the test substance to translocate across the epithelium (Bouwmeester et al. 2011).

Apart from Ag NMs, a number of further metal and metal oxide NMs have been studied in repeated-dose oral toxicity studies: At 537 mg/kg, zinc oxide *NMs* (40 nm, TEM and SEM; 202 nm, photon correlation spectroscopy), administered orally to rats for 90 days, resulted in anemia-related hematological changes, mild to moderate pancreatitis, and, in the male rats, reduced weight gain (Seok et al. 2013). In mice, 14-day oral exposure to 300 mg/kg ZnO NMs (30 nm, TEM; 272 nm, DLS) resulted in liver damage (determined by clinical chemistry and histopathology) and DNA damages in the liver and kidney (determined by comet assay; Sharma et al. 2012).

At 1,000 mg/kg, 28-day oral exposure of Fe_2_O_3_ NMs (30 nm, TEM; 363 nm, DLS and Laser Doppler Velocimetry) induced damages of the liver and kidneys in rats, as observed by histopathology and clinical chemistry. Furthermore, synaptic transmission and nerve conduction were affected in the animals’ brains, however, without corresponding histopathological changes (Kumari et al. 2012). Upon 28-day oral exposure, 300 and 1,000 mg/kg manganese oxide NMs (43 nm, TEM; 325 nm, DLS) induced genetic damage in the leukocytes and bone marrow of rats, and alterations in liver, spleen, kidney, and brain (determined by histopathology and clinical chemistry; Singh et al. 2013).

In rats, 5-day oral exposure to 50–200 mg/kg Copper NMs (primary size: 25 nm; size distribution 5–60 nm, DLS and atomic force microscopy) resulted in widespread renal proximal tubule necrosis (Liao and Liu 2012). In mice, 14-day oral exposure to 20 mg/kg ‘biogenic’ (i.e., produced by *Bacillus sp.)* selenium NMs (105–130 nm, TEM; no characterization of prepared test substance) caused lower body weight and changes in hematological parameters and clinical chemistry (Shakibaie et al. 2013).

Five-day oral exposure of rats to 1 or 2 mg/kg anatase TiO_2_ NMs (<25 nm, TEM; agglomeration up to 1.6 µm, SEM) induced histological alterations in female animals in the spleen, thyroid, adrenal medulla, adrenal cortex, and ovarian granulosa, albeit without corresponding clinical signs of toxicity. In male rats, thyroid function was altered and testosterone levels were increased in the high-dose group (Tassinari et al. 2014). Bu et al. (2010) exposed rats orally to 1 g/kg rutile-anatase TiO_2_ NMs (<50 nm, XRD; no information on hydrodynamic particle diameter) for 14 consecutive days and observed disturbances in the amino acid and energy metabolisms, in the gut microflora environment, as well as slight injury to the heart and liver tissues.

To date, in vivo studies comparing the effects of different nanomaterials under identical experimental conditions or assessing organ burden upon oral NM administration are scarce. An exception is the study by Cho et al. (2013) who exposed rats orally to 260–1,042 mg/kg TiO_2_ (80 % anatase, 20 % rutile; 26 nm, SEM, 38 nm, DLS) or 134–536 mg/kg ZnO NMs (90 nm, SEM; 202 nm, DLS) for 13 weeks (7 days/week). Ti or Zn organ tissue burden was assessed upon necropsy revealing that TiO_2_ nanoparticles were not significantly increased in sampled organs, even in the group receiving the highest dose (1,042 mg/kg). By contrast, Zn concentrations in the liver and kidney were significantly increased, and minimally increased in the spleen and brain, as compared to the vehicle controls (Cho et al. 2013).

In regard to carbon-based NMs, there were no treatment-related effects in rats upon 28-day oral exposure to 12.5 mg/kg single-walled carbon nanotubes (SWCNTs; basic morphology sheet form consisting of entangled CNTs, diameter 2 nm) or 50 mg/kg multi-walled carbon nanotubes (MWCNTs; basic morphology entangled CNTs, diameter 30 nm; Matsumoto et al. 2012). Nevertheless, Matsumoto et al. conclude that, due to CNT absorption and persistence, longer-term effects of these materials cannot be excluded. Finally, upon 29-day oral exposure to 1,000 mg/kg *fullerene C60* (0.71 nm diameter; no further characterization), liver and spleen weights were slightly increased in rats without corresponding signs of toxicity (Takahashi et al. 2012).

In summary, reports on repeated-dose oral toxicity studies investigating NM effects are scarce. Oftentimes, they were not conducted in accordance with standardized test guidelines, or the test substances were not submitted to comprehensive characterization in the prepared states. Furthermore, comparative hazard assessments of different NMs under identical experimental conditions are lacking, just as investigations on the reversibility or progression of effects. Many effects were observed by histopathological examination or clinical chemistry, but were not accompanied by corresponding clinical findings. Therefore, it is oftentimes difficult, if not impossible, to determine the relevance such adverse effects, and it is not yet possible to come to a conclusion in regard to, e.g., ranking of toxic effects of different types of NMs upon subacute or sub-chronic oral exposure. Of note, however, of the seven NMs tested in the present study, only SiO_2·_naked elicited adverse effects upon 5-day inhalation, but only locally in the lungs, and this effect was only observed at the highest test substance concentration. Since inhalation is the most important route of exposure for NMs, these findings corroborate the assumption that the tested NMs do not elicit substantial adverse effects upon the experimental conditions applied in the present study.

### Metabolome analysis

Likewise, under the conditions of the present study, the seven SiO_2_, ZrO_2_, and BaSO_4_ NMs did not affect the plasma metabolome in female or male rats. In accordance with the criteria defined by ECETOC to determine adverse effects based on ‘-omics’ data (ECETOC 2008, 2010, 2013) and since there were no matches with the specific toxicity patterns of the MetaMap^®^Tox database, the dose level tested, i.e., 1,000 mg/kg, can be considered as metabolomic no-observed-adverse-effect level (NOAEL) upon oral 28-day exposure to rats. Furthermore, the number of significant endogenous metabolite changes was below or at the false positive rate for all treatments in both sexes. Therefore, the dose level of 1,000 mg/kg can also be considered as a metabolomic no-observed-effect level (NOEL).

To date, only few studies have performed metabolic profiling of NM effects (Schnackenberg et al. 2012). There was no metabolomics study reports to be found in the peer-reviewed literature allowing comparing the results of the present study to those from similar investigations. Those NM metabolomics studies retrieved not only investigated different types of NMs, which sometimes further were not submitted to a thorough physico-chemical characterization. These studies also applied different forms of test substance administration and evaluated *different tissues or body fluids in mice or rats* by different technologies and procedures for metabolic profiling. In this context, LC–MS/MS and GC–MS techniques have been assessed as being more sensitive than nuclear magnetic resonance (NMR) techniques, thereby increasing the likelihood of detecting relevant biomarkers or patterns of change (van Ravenzwaay et al. 2007; Schnackenberg et al. 2012). A further issue to be taken into account in assessing the relevance of metabolomics studies is the statistical procedure applied in analyzing changes in metabolite patterns (ECETOC, 2008, 2010; Schnackenberg et al. 2012). All of these differences in testing schemes make the results of currently available metabolomics studies difficult, if not impossible to compare.

In the *liver tissues and serum of mice* that were killed 3 h after intravenous injection of SiO_2_ NMs (30, 70, and 300 nm; DLS), changes in metabolite patterns were revealed by GC–MS combined with pattern recognition approaches, indicating disturbances in energy metabolism as well as amino, lipid, and nucleotide metabolism, which were considered attributable to hepatotoxicity (Lu et al. 2011).

Applying 150 µg amorphous SiO_2_ NMs (10 and 80 nm, TEM; no secondary particle characterization) by intranasal instillation into both nostrils of *rats* for 90 days, the metabolomic profile of the *serum*, determined by NMR, exhibited significantly increased lactate, alanine, acetate, creatine, and choline coupled with a considerable decrease in the glucose level (Parveen et al. 2012). Parveen et al. conclude that these observations implicate an impairment of the tricarboxylic acid cycle and liver metabolism, suggesting that SiO_2_ NMs may potentially induce hepatotoxicity in rats upon sub-chronic administration.

In an in vitro study with *human MRC*-*5 fetal lung fibroblasts* treated with up to 80.0 μg mL SiO_2_ NMs (average diameter 45 nm; no indication of secondary particle characterization), Huang et al. (2012) recorded dose-dependent decreases of various cellular amino acid levels as well as glutathione accompanied with increased phospholipid and urea concentrations (making use of GC–MS and LC–MS). However, there was no evidence of morphological or cell viability changes (as determined by light microscopy and in a tetrazolium reduction assay; Huang et al. 2012).

In the *urine and serum of rats* exposed orally to 1 g/kg rutile-anatase TiO_2_ NMs (<50 nm, XRD; no information on hydrodynamic particle diameter) for 14 days, Bu et al. (2010) observed metabolite changes indicating disturbances in the energy and amino acid metabolism by NMR using principal components and partial least squares discriminant analysis. After intratracheal instillation of *rats* to 0.8–20 mg/kg TiO_2_ (5 nm; TEM, no indication of secondary particle characterization), *urine* metabolite changes reflecting acute, transient metabolic disturbances of the liver and kidneys were only observed in the low-dose groups (Tang et al. 2010). These changes were not paralleled by histopathological or serum biochemical alterations, whereas at higher dosages, the TiO_2_ NMs provoked histopathological pulmonary inflammatory responses. Tang and co-authors conclude that at low dosages, TiO_2_ NMs can enter the blood circulation, whereas at higher dosages, the particles tend to agglomerate and accumulate in the lung (Tang et al. 2010).

Hadrup et al. (2012b) applied high performance liquid chromatography–quadrupole time-of-flight mass spectrometry (HPLC–QTOF–MS) and principal component analysis (PCA) to determine *rat urine* metabolite patterns after 18-day oral exposure of up to 9.0 mg/kg PVP-coated Ag NMs (14 nm; DLS). Thereby, Hadrup et al. observed metabolomic differences in the urine from female, but not male rats. Metabolomic differences between male and females have been described before (van Ravenzwaay et al. 2007). Administration of nanoform silver increased both uric acid and its degradation product allantoin, suggesting that purine metabolism was affected (Hadrup et al. 2012b).

Feng et al. (2010) investigated the metabolic responses induced by 25 mmol/L of uncoated and dextran-coated ultra-small superparamagnetic iron oxide (2.3–4 nm; hydrodynamic particle size: 20 nm; no indication of characterization methodology) upon intravenous injection into *rats*. Applying NMR-based analysis in combination with multivariate statistical analysis of both *rat urine and plasma* metabolomes, Feng and co-authors observed size- and surface chemistry-dependent changes in energy, lipid, glucose, and amino acid metabolic pathways that may be attributed to disturbances of hepatic, renal, and cardiac functions.

## Conclusion

Under the experimental conditions of the present study, neither the 4 SiO_2_ NMs (the core material and its 3 variants with different surface functionalizations), nor the 2 surface-functionalized ZrO_2_ NMs or BaSO_4_ NM-220, elicited any effects upon 28-day oral exposure to rats that were assessed as test substance-related and adverse. Similarly, apart from the core SiO_2·_naked also none of the test materials induced adverse effects upon 5-day inhalation exposure to rats (Landsiedel et al. 2014). These findings—together with the lack of changes in metabolite patterns recorded in the present study—suggest that the tested substances do not elicit systemic toxicity under the reported experimental conditions. Further investigations, however, are necessary to develop and assess standardized procedures for test substance preparation for oral administration ensuring reliable substance homogeneity. Likewise, further investigations should aim at determining the amount of NM that can enter the body upon ingestion under different experimental conditions taking into account a variety of different test substance preparations.

To date, only a limited number of subacute and sub-chronic toxicity studies investigating NM effects upon oral exposure are available, and the variety of different NMs as well as of different testing protocols applied makes it difficult to compare the outcome of different studies. Due to the diversity and complexity of different types of NMs, several authors have highlighted that conducting repeated-dose toxicity studies in rodents for every new NM introduced onto the market is impractical, if not impossible (Lai 2012; Nel et al. 2013; Oomen et al. 2014). Therefore, further work should also aim at making use of the data collected so far to develop and implement concern-driven integrated strategies for the testing and assessment of nanomaterials taking into account realistic exposure scenarios (Lai 2012; Nel et al. 2013; Oomen et al. 2014).

## Electronic supplementary material

Below is the link to the electronic supplementary material.

Supplementary material 1 (DOCX 124 kb)

Supplementary material 2 (PDF 59 kb)

Supplementary material 3 (PDF 47 kb)

Supplementary material 4 (PDF 60 kb)

## Footnotes

1 Note: Except otherwise indicated, all test substance concentrations for oral exposure expressed as ‘mg/kg’ refer to mg/kg body weight (bw) per day.

## References

[CR1] Aninwene GE, Stout D, Yang Z, Webster TJ (2013). Nano-BaSO_4_: a novel antimicrobial additive to pellethane. Int J Nanomed.

[CR2] Anon (2006) Regulation (EC) No 1907/2006 of the European Parliament and of the Council of 18 December 2006 concerning the Registration, Evaluation, Authorisation and Restriction of Chemicals (REACH), establishing a European Chemicals Agency, amending Directive 1999/45/EC and repealing Council Regulation (EEC) No 793/93 and Commission Regulation (EC) No 1488/94 as well as Council Directive 76/769/EEC and Commission Directives 91/155/EEC, 93/67/EEC, 93/105/EC and 2000/21/EC. OJ L 396:1, 30 December 2006

[CR3] Anon (2012) Commission staff working paper accompanying the Communication from the Commission to the European Parliament, the Council and the European Economic and Social Committee on the second regulatory review on nanomaterials: Types and uses of nanomaterials, including safety aspects (COM(2012) 572 final. SWD(2012) 288 final. Brussels, 3 October 2012

[CR4] Bouwmeester H, Poortman J, Peters RJ, Wijma E, Kramer E, Makama S, Puspitaninganindita S, Marvin HJP, Peijnenburg AACM, Hendriksen PJM (2011). Characterization of translocation of silver nanoparticles and effects on whole-genome gene expression using an in vitro intestinal epithelium coculture model. ACS Nano.

[CR5] Bu Q, Yan G, Deng P, Peng F, Lin H, Xu Y, Cao Z, Zhou T, Xue A, Wang Y, Cen X, Zhao YL (2010). NMR-based metabonomic study of the sub-acute toxicity of titanium dioxide nanoparticles in rats after oral administration. Nanotechnol.

[CR6] Cho WS, Kang BC, Lee JK, Jeong J, Che JH, Seok SH (2013). Comparative absorption, distribution, and excretion of titanium dioxide and zinc oxide nanoparticles after repeated oral administration. Part Fibre Toxicol.

[CR7] Coco R, Plapied L, Pourcelle V, Jérôme C, Brayden DJ, Schneider YJ, Préat V (2013). Drug delivery to inflamed colon by nanoparticles: comparison of different strategies. Int J Pharm.

[CR8] Dekkers S, Bouwmeester H, Bos PMJ, Peters RJB, Rietveld AG, Oomen AG (2013). Knowledge gaps in risk assessment of nanosilica in food: evaluation of the dissolution and toxicity of different forms of silica. Nanotoxicol.

[CR9] Delie F (1998). Evaluation of nano- and microparticle uptake by the gastrointestinal tract. Adv Drug Deliv Rev.

[CR10] ECETOC (2002) Recognition of, and differentiation between, adverse and non-adverse effects in toxicology studies. Technical Report No. 85. ISSN-0773-6347-85, Brussels, December 2002

[CR11] ECETOC (2008) WR 11. Workshop on the application of ‘omics’ in toxicology and ecotoxicology: case studies and risk assessment. Brussels, July 2008. http://www.ecetoc.org/workshop-reports

[CR12] ECETOC (2010) WR 19. Omics in (eco)toxicology: case studies and risk assessment. June 2010. http://www.ecetoc.org/workshop-reports

[CR13] ECETOC (2013) WR 25. ‘Omics and risk assessment science. October 2013. http://www.ecetoc.org/workshop-reports

[CR14] ECHA (2012) Guidance on information requirements and chemical safety assessment. Appendix R7-1 Recommendations for nanomaterials applicable to Chapter R7a Endpoint specific guidance. European Chemicals Agency ECHA-12-G-03-EN, April 2012. 60 pp. http://echa.europa.eu/documents/10162/13632/appendix_r7a_nanomaterials_en.pdf

[CR15] EFSA (2009) Scientific opinion on the potential risks arising from nanoscience and nanotechnologies on food and feed safety. Response to question EFSA-Q-2007-124a. 10 February 2009. EFSA J 958:1–39. http://www.efsa.europa.eu/en/efsajournal/doc/1052.pdf

[CR16] EFSA (2011) Scientific opinion: guidance on the risk assessment of the application of nanoscience and nanotechnologies in the food and feed chain. 10 May 2011. EFSA J 9:2140. http://www.efsa.europa.eu/de/efsajournal/pub/2140.htm 10.2903/j.efsa.2018.5327 PMC7009542 32625968

[CR17] Fallingborg J (1999). Intraluminal pH of the human gastrointestinal tract. Dan Med Bull.

[CR18] Feng J, Liu H, Zhang L, Bhakoo K, Lu L (2010). An insight into the metabolic responses of ultra-small superparamagnetic particles of iron oxide using metabonomic analysis of biofluids. Nanotechnol.

[CR19] Florence AT (1997). The oral absorption of micro- and nanoparticulates: neither exceptional nor unusual. Pharmaceut Res.

[CR20] Florence AT (2005). Nanoparticle uptake by the oral route: fulfilling its potential?. Drug Discovery Today: Technol.

[CR21] Fröhlich E, Roblegg E (2012). Models for oral uptake of nanoparticles in consumer products. Toxicol.

[CR22] Gad SC, Cassidy CD, Aubert N, Spainhour B, Robbe H (2006). Nonclinical vehicle use in studies by multiple routes in multiple species. Int J Toxicol.

[CR23] Hadrup N, Lam HR (2014). Oral toxicity of silver ions, silver nanoparticles and colloidal silver—a review. Regul Toxicol Pharmacol.

[CR24] Hadrup N, Loeschner K, Bergström A, Wilcks A, Gao X, Vogel U, Frandsen HL, Larsen EH, Lam HR, Mortensen A (2012). Subacute oral toxicity investigation of nanoparticulate and ionic silver in rats. Arch Toxicol.

[CR25] Hadrup N, Lam HR, Loeschner K, Mortensen A, Larsen EH, Frandsen H (2012). Nanoparticulate silver increases uric acid and allantoin excretion in rats, as identified by metabolomics. J Appl Toxicol.

[CR26] Hankin SM, Peters SAK, Poland CA, Foss Hansen S, Holmqvist J, Ross BL, Varet J, Aitken RJ (2011) Specific advice on fulfilling information requirements for nanomaterials under REACH (RIP-oN 2)—final project peport. REACH-NANO consultation. RNC/RIP-oN2/FPR/1/FINAL. 356 pp. http://ec.europa.eu/environment/chemicals/nanotech/pdf/report_ripon2.pdf

[CR27] Higashisaka K, Yoshioka Y, Yamashita K, Morishita Y, Fujimura M, Nabeshi H, Nagano K, Abe Y, Kamada H, Tsunoda S, Yoshikawa T, Itoh N, Tsutsumi Y (2011). Acute phase proteins as biomarkers for predicting the exposure and toxicity of nanomaterials. Biomat.

[CR28] Hillyer JF, Albrecht RM (2001). Gastrointestinal persorption and tissue distribution of differently sized colloidal gold nanoparticles. J Pharm Sci.

[CR29] Hong JS, Kim S, Lee SH, Jo E, Lee B, Yoon J, Eom IC, Kim HM, Kim P, Choi K, Lee MY, Seo YR, Kim Y, Lee Y, Choi J, Park K (2014). Combined repeated-dose toxicity study of silver nanoparticles with the reproduction/developmental toxicity screening test. Nanotoxicol.

[CR30] Huang SM, Zuo X, Li JJ, Li SF, Bay BH, Ong CN (2012). Metabolomics studies show dose-dependent toxicity induced by SiO_2_ nanoparticles in MRC-5 human fetal lung fibroblasts. Adv Health Mater.

[CR31] Kamp H, Buesen R, Fabian E, Herold M, Krennrich G, Leibold E, Looser R, Mellert W, Nishino T, Prokoudine A, Strauss V, Walk T, Wiemer J, van Ravenzwaay B (2010) Metabolite profiling in rat plasma as a potential new tool for the assessment of chemically induced toxicity. In: DFG. Risk Assessment of Phytochemicals in Food. Wiley, Weinheim, ISBN 978-3-527-32929-8

[CR32] Kamp H, Strauss V, Wiemer J, Leibold E, Walk T, Mellert W, Looser R, Prokoudine A, Fabian E, Krennrich G, Herold M, van Ravenzwaay B (2012). Reproducibility and robustness of metabolome analysis in rat plasma of 28-day repeated dose toxicity studies. Toxicol Lett.

[CR33] Kamp H, Fabian E, Groeters S, Herold M, Krennrich G, Looser R, Mattes W, Mellert W, Prokoudine A, Ruiz-Noppinger P, Strauss V, Walk T, Wiemer J, van Ravenzwaay B (2012). Application of in vivo metabolomics to preclinical/toxicological studies: case study on phenytoin-induced systemic toxicity. Bioanalysis.

[CR34] Kim YS, Kim JS, Cho HS, Rha DS, Kim JM, Park JD, Choi BS, Lim R, Chang HK, Chung YH, Kwon IH, Jeong J, Han BS, Yu IJ (2008). Twenty-eight-day oral toxicity, genotoxicity, and gender-related tissue distribution of silver nanoparticles in Sprague-Dawley rats. Inhal Toxicol.

[CR35] Kim YS, Song MY, Park JD, Song KS, Ryu HR, Chung YH, Chang HK, Lee JH, Oh KH, Kelman BJ, Hwang IK, Yu IJ (2010). Subchronic oral toxicity of silver nanoparticles. Part Fibre Toxicol.

[CR36] Kuhlbusch TA, Krug HF, Nau K (2009). Nanocare—health related effects of nanoparticles. Final scientific report.

[CR37] Kumari M, Rajak S, Singh SP, Kumari SI, Kumar PU, Murty USN, Mahboob M, Grover P, Rahman MF (2012). Repeated oral dose toxicity of iron oxide nanoparticles: biochemical and histopathological alterations in different tissues of rats. J Nanosci Nanotechnol.

[CR38] Lai DY (2012). Toward toxicity testing of nanomaterials in the 21st century: a paradigm for moving forward. Wiley Interdiscip Rev Nanomed Nanobiotechnol.

[CR39] Landsiedel R, Ma-Hock L, Haussmann HJ, van Ravenzwaay B, Kayser M, Wiench K (2012). Inhalation studies for the safety assessment of nanomaterials: status quo and the way forward. Wiley Interdiscip Rev Nanomed Nanobiotechnol.

[CR40] Landsiedel R, Fabian E, Ma-Hock L, Wohlleben W, Wiench K, Oesch F, van Ravenzwaay B (2012). Toxico-/biokinetics of nanomaterials. Arch Toxicol.

[CR41] Landsiedel R, Ma-Hock L, Hofmann T, Wiemann M, Strauss V, Treumann S, Wohlleben W, Gröters S, Wiench K, van Ravenzwaay B (2014). Application of short-term inhalation studies to assess the inhalation toxicity of nanomaterials. Part Fibre Toxicol.

[CR42] Liao MY, Liu HG (2012). Gene expression profiling of nephrotoxicity from copper nanoparticles in rats after repeated oral administration. Env Toxicol Pharmacol.

[CR43] Loeschner K, Hadrup N, Qvortrup K, Larsen A, Gao X, Vogel U, Mortensen A, Lam HR, Larsen EH (2011). Distribution of silver in rats following 28 days of repeated oral exposure to silver nanoparticles or silver acetate. Part Fibre Toxicol.

[CR44] Lu X, Tian Y, Zhao Q, Jin T, Xiao S, Fan X (2011). Integrated metabonomics analysis of the size-response relationship of silica nanoparticles-induced toxicity in mice. Nanotechnol.

[CR45] Matsumoto M, Serizawa H, Sunaga M, Kato H, Takahashi M, Hirata-Koizumi M, Ono A, Kamata E, Hirose A (2012). No toxicological effects on acute and repeated oral gavage doses of single-wall or multi-wall carbon nanotubes in rats. J Toxicol Sci.

[CR46] Nagano T, Yoshioka Y, Higashisaka K, Kunieda A, Hata K, Nagano K, Abe Y, Kamada H, Tsunoda S, Nabeshi H, Yoshikawa T, Tsutsumi Y (2012). Potential of acute-phase proteins as biomarkers for sub-nano platinum exposure. Pharmazie.

[CR47] Nel AE, Xia T, Meng H, Wang X, Lin S, Ji Z, Zhang H (2013). Nanomaterial toxicity testing in the 21^st^ century: use of a predictive toxicological approach and high-throughput screening. Acc Chem Res.

[CR48] OECD (2008a) List of manufactured nanomaterials and list of endpoints for phase one of the OECD testing programme. Series on the safety of manufactured nanomaterials, number 6. ENV/JM/MONO(2008)13/REV, OECD, Paris, 7 July 2008

[CR49] OECD (2008b) Repeated-dose 28-day oral toxicity study in rodents. OECD guidelines for the testing of chemicals No. 407. OECD, Paris, 3 October 2008

[CR50] OECD (2010) Guidance manual for the testing of manufactured nanomaterials: OECD’s sponsorship programme; first revision. ENV-JM-MONO(2009)20-REV, OECD, Paris, 2 June 2010

[CR51] Oomen AG, Bos PMJ, Fernandes TF, Hund-Rinke K, Boraschi D, Byrne HJ, Aschberger K, Gottardo S, van der Kammer F, Kühnel D, Hristozov D, Marcomini A, Migliore L, Scott-Fordsmand J, Wick P, Landsiedel R (2014). Concern-driven integrated approaches to nanomaterial testing and assessment—Report of the NanoSafety Cluster Working Group 10. Nanotoxicol.

[CR52] Park E-J, Bae E, Yi J, Kim Y, Choi K, Lee SH, Yoon J, Lee BC, Park K (2010). Repeated-dose toxicity and inflammatory responses in mice by oral administration of silver nanoparticles. Env Toxicol Pharmacol.

[CR53] Parveen A, Rizvi SH, Gupta A, Singh R, Ahmad I, Mahdi F, Mahdi AA (2012). NMR-based metabonomics study of sub-acute hepatotoxicity induced by silica nanoparticles in rats after intranasal exposure. Cell Mol Biol.

[CR54] Ricker A, Liu-Snyder P, Webster TJ (2008). The influence of nano MgO and BaSO_4_ particle size additives on properties of PMMA bone cement. Int J Nanomed.

[CR55] RIP-oN 1 (2011) REACH implementation project. Substance identification of nanomaterials. Advisory Report. AA N° 070307/2009/DI/534733 between DG ENV and JRC. European Commission, Joint Research Centre, Institute for Health and Consumer Protection. 76 pp. http://ec.europa.eu/environment/chemicals/nanotech/pdf/report_ripon1.pdf

[CR56] Savolainen K, Backman U, Brouwer D, Fadeel B, Fernandes T, Kuhlbusch T, Landsiedel R, Lynch I, Pylkkänen L (2013) Nanosafety in Europe 2015–20125: towards safe and sustainable nanomaterials and nanotechnology innovations. Finnish Institute of Occupational Health, Finland. 218 pp. www.ttl.fi/en/publications/electronic_publications/pages/default.aspx

[CR57] Schnackenberg LK, Sun J, Beger RD (2012). Metabolomics techniques in nanotoxicology studies. Methods Mol Biol.

[CR58] Seok SH, Cho W-S, Park JS, Na Y, Jang A, Kim H, Cho Y, Kim T, You J-R, Ko S, Kang B-C, Lee JK, Jeong J, Che J-H (2013). Rat pancreatitis produced by 13-week administration of zinc oxide nanoparticles: biopersistence of nanoparticles and possible solutions. J Appl Toxicol.

[CR59] Shakibaie M, Shahverdi AR, Faramarzi MA, Hassanzadeh GR, Rahimi HR, Sabzevari O (2013). Acute and subacute toxicity of novel biogenic selenium nanoparticles in mice. Pharmaceut Biol.

[CR60] Sharma V, Singh P, Pandey AK, Dhawan A (2012). Induction of oxidative stress, DNA damage and apoptosis in mouse liver after sub-acute oral exposure to zinc oxide nanoparticles. Mutat Res.

[CR61] Singh SP, Kumari M, Kumari SI, Rahman MF, Mahboob M, Grover P (2013). Toxicity assessment of manganese oxide micro and nanoparticles in Wistar rats after 28 days of repeated oral exposure. J Appl Toxicol.

[CR62] Hellak B, Hülser T, Izak E, Kuhlbusch T, Meyer F, Spree, M, Voetz M, Wiggers H, Wohlleben W (2012) NanoGEM deliverable 1.3.1: Characterization report for all nanoGEM materials. M24, 41 pp. http://www.nanogem.de/cms/nanogem/upload/Veroeffentlichungen/nanoGEM_Del1.3.1_Characterization_Materials_2013_04_24.pdf

[CR63] Takahashi M, Kato H, Doi Y, Hagiwara A, Hirata-Koizumi M, Ono A, Kubota R, Nishimura T, Hirose A (2012). Sub-acute oral toxicity study with fullerene C60 in rats. J Toxicol Sci.

[CR64] Tang M, Zhang T, Xue Y, Wang S, Huang M, Yang Y, Lu M, Lei H, Kong L, Yuepu P (2010). Dose dependent in vivo metabolic characteristics of titanium dioxide nanoparticles. J Nanosci Nanotechnol.

[CR65] Tassinari R, Cubadda F, Moracci G, Aureli F, D’Amato M, Valeri M, De Berardis B, Raggi A, Mantovani A, Passeri D, Rossi M, Maranghi F (2014). Oral, short-term exposure to titanium dioxide nanoparticles in Sprague–Dawley rat: focus on reproductive and endocrine systems and spleen. Nanotoxicol.

[CR66] van der Zande M, Vandebriel RJ, van Doren E, Kramer E, Herrera Rivera Z, Serrano-Rojero CS, Gremmer ER, Mast J, Peters RJB, Hollman PCH, Hendriksen PJM, Marvin HJP, Peijnenburg AACM, Bouwmeester H (2012). Distribution, elimination, and toxicity of silver nanoparticles and silver ions in rats after 28-day oral exposure. ACS Nano.

[CR67] van der Zande M, Vandebriel RJ, Groot MJ, Kramer E, Rivera ZH, Rasmussen K, Ossenkoppele JS, Tromp P, Gremmer ER, Peters RJB, Hendriksen PJ, Marvin HJP, Peijnenburg AACM, Bouwmeester H (2014). Sub-chronic toxicity study in rats orally exposed to nanostructured silica. Part Fibre Toxicol.

[CR68] van Ravenzwaay B, Cunha GC-P, Leibold E, Looser R, Mellert W, Prokoudine A, Walk T, Wiemer J (2007). The use of metabolomics for the discovery of new biomarkers of effect. Toxicology Lett.

[CR69] van Ravenzwaay B, Coelho-Palermo Cunha G, Fabian E, Herold M, Kamp H, Krennrich G, Krotzky A, Leibold E, Looser R, Mellert W, Prokoudine A, Strauss V, Trethewey R, Walk T, Wiemer J, Cho WCS (2010). The use of metabolomics in cancer research. An omics perspective of cancer.

[CR70] van Ravenzwaay B, Coelho-Palermo Cunha G, Strauss V, Wiemer J, Leibold E, Kamp H, Walk T, Mellert W, Looser R, Prokoudine A, Fabian E, Krennrich G, Herold M (2010). The individual and combined metabolite profiles (metabolomics) of dibutylphthalate and di(2-ethylhexyl)phthalate following a 28-day dietary exposure in rats. Toxicology Lett.

[CR71] van Ravenzwaay B, Herold M, Kamp H, Kapp MD, Fabian E, Looser R, Krennrich G, Mellert W, Prokoudine A, Strauss V, Walk T, Wiemer J (2012). Metabolomics: a tool for early detection of toxicological effects and an opportunity for biology based grouping of chemicals-from QSAR to QBAR. Mutat Res.

[CR72] van Ravenzwaay B, Brunborg G, Kleinjans J, Galay Burgos M, Vrijhof H (2012). Use of ‘omics to elucidate mechanism of action and integration of ‘omics in a systems biology concept. Mutat Res.

[CR73] Wohlleben W (2012). Validity range of centrifuges for the regulation of nanomaterials: from classification to as-tested coronas. J Nanopart Res.

[CR74] Wohlleben W, Ma-Hock L, Boyko V, Cox G, Egenolf H, Freiberger H, Hinrichsen B, Hirth S, Landsiedel R (2013). Nanospecific guidance in REACH: a comparative physicalchemical characterization of 15 materials with methodical correlations. J Ceramic Sci Technol.

[CR75] Wohlleben W, Kuhlbusch T, Lehr CM, Schnekenburger J (eds) (2014) Safety of nanomaterials along their lifecycle: release, exposure and human hazards. Taylor & Francis Oxford, New York, ISBN 978-1-46-656786-3, (in print)

