# Supplementary material for: Effects of SiO2, ZrO2, and BaSO4 nanomaterials with or without surface functionalization upon 28-day oral exposure to rats
Source: Arch Toxicol. 2014 Aug 28;88(10):1881–906. doi: 10.1007/s00204-014-1337-0 (PMC4161931; doi:10.1007/s00204-014-1337-0)
Supplement: Supplementary file 1 — Supplementary material 1 (DOCX 124 kb) [file 204_2014_1337_MOESM1_ESM.docx]

**Supplementary Information to:**

**Effects of SiO_2_, ZrO_2_, and BaSO_4_ nanomaterials with or without surface functionalization upon 28-day oral exposure to rats**

*Buesen, R., Landsiedel, R., Sauer, U.G., Wohlleben, W., Strauss, V., Groeters, S., Kamp, H., van Ravenzwaay, B.*

**Study performance, details**

*Detailed clinical observations, heath checks, assessment of food and water consumption and determination of body weight*

Detailed clinical observations (DCOs), adhering to the parameters listed in OECD TG 407, paragraph 26, were performed on all animals prior to the administration period and thereafter at weekly intervals. Prior to the first DCO, the male and female animals were each assigned to the individual test groups. The list of randomization instructions was compiled with a computer ensuring that the overall weight variation of the animals did not exceed 20% of the mean weight of each sex. For all DCOs, the rats were transferred to an open field (50 × 37.5 cm of size, with sides of 25 cm height). Again, any findings were ranked according to their degree of severity, if applicable.

A check for moribund and dead animals was made twice daily on working days and once daily on Saturdays, Sundays and public holidays. Additionally, each day shortly before test substance administration, all animals were observed for any abnormal clinical signs. They were further observed within 2 and 5 hours after the administration. For each animal, the occurrence of any abnormal clinical signs was recorded. Findings were ranked according to their degree of severity, if applicable.

Food consumption was determined weekly over a period of 1 day and calculated as mean food consumption in grams per animal and day. Similarly, drinking water consumption was determined weekly over a period of 4 days and calculated as mean water consumption in grams per animal and day. For all animals, also the body weight was determined weekly, and test substance dosages related to the most recently measured body weight.

*Blood sampling, assessment of hematological parameters and clinical chemistry*

For blood sampling, the retro-bulbar venous plexus from fasted, anaesthetized animals was punctuated in the mornings (anesthetics: isoflurane, Essex GmbH, Munich, Germany).

- The hematological parameters were determined in blood supplemented with EDTA‑K_3_ as anticoagulant using a hematology instrument (ADVIA 120 Siemens Healthcare Diagnostics, Germany). Prothrombin time (Hepatoquick^®^ Test (Stago, Duesseldorf, Germany)) was assessed in blood with Na-citrate additive using a coagulometer (AMAX Destiny Plus, Trinity Biotech, Lemgo, Germany).
- An automatic analyzer (Hitachi 917; Roche, Mannheim, Germany) was used to examine the clinical chemical parameters.
- Haptoglobin and α2-macroglobulin were measured in enzyme-linked immunosorbent assays (ELISA) produced by Immunology Consultants Laboratory Inc., Newberg, OR, USA (catalogue numbers E-25A2M and E-25HPT, respectively). Both ELISAs were evaluated with a Sunrise™ MTP Reader (Tecan AG, Maennedorf, Switzerland) using the Magellan Software provided by the instrument producer.
- For the troponin I measurements, serum samples were sent to the Institute for Preclinical Drug Safety, ALTANA Pharma, Hamburg, Germany. Cardiac troponin I was determined in a direct chemiluminescence immunoassay using an ADVIA Centaur^®^ CP, Siemens Healthcare Diagnostics, Eschborn, Germany.

Towards the end of the administration period, urine was collected overnight with concurrent food and water withdrawal. For urinalysis, the individual animals were transferred to metabolism cages.

- Urine volume was determined with graduated tubes, its color and turbidity were assessed by visual inspection, the sediment microscopically, and the specific gravity with a refractometer (Atago Co., Ltd., Tokyo, Japan).
- Urine constituents were determined semi-quantitatively making use of test strips (Combur‑10 Test^®^, Roche, Mannheim, Germany) evaluating the dry chemical reactions with a reflection photometer (Miditron M; Roche, Mannheim, Germany).
