# Supplementary material for: Effects of SiO2, ZrO2, and BaSO4 nanomaterials with or without surface functionalization upon 28-day oral exposure to rats
Source: Arch Toxicol. 2014 Aug 28;88(10):1881–906. doi: 10.1007/s00204-014-1337-0 (PMC4161931; doi:10.1007/s00204-014-1337-0)
Supplement: Supplementary file 2 — Supplementary material 2 (PDF 59 kb) [file 204_2014_1337_MOESM2_ESM.pdf]

HISTORICAL CONTROL DATA OF CLINICAL PATHOLOGY TESTING [1)]

Species: Rat
Sex: Female
Strain: Wistar
Age: 10 weeks
Study period: 4 weeks
Fasting before bloo 16 hours
Anaesthesia Isoflurane
Data print out at 5-Aug-14
Clinical Chemistry I Hitachi 917, Roche
Haematology Analy ADVIA 120, Bayer
Coagulation Analyz AMAX Destiny Plus, Trinity

Parameter:	WBC	RBC	HGB	HCT	MCV	MCH	MCHC	PLT	RETI	NEUT	LYMPH	MONO	EOS	BASO	LUC	NEUT	LYMPH	MONO	EOS	BASO	LUC	HQT	ALT
Unit:	GIGA/L	TERA/L	MMOL/L	L/L	FL	FMOL	MMOL/L	GIGA/L	%	%	%	%	%	%	%	GIGA/L	GIGA/L	GIGA/L	GIGA/L	GIGA/L	GIGA/L	SECONDS	MYKAT/L
Study No,																							
07088	4,26	7,32	8,7	0,370	50,5	1,19	23,45	835	1,9	9,3	86,8	1,2	1,7	0,6	0,4	0,40	3,69	0,05	0,07	0,02	0,02	34,9	0,66
07080	3,95	7,48	8,6	0,374	50,0	1,15	22,93	860	1,7	12,2	82,4	2,3	1,8	0,8	0,5	0,46	3,27	0,09	0,07	0,03	0,02	35,0	0,59
07105	4,31	7,48	9,1	0,374	50,0	1,22	24,34	895	2,2	7,9	88,2	1,2	2,4	0,0	0,3	0,35	3,79	0,05	0,10	0,00	0,02	33,0	0,68
07104	4,28	7,42	8,7	0,376	50,7	1,18	23,18	954	2,6	11,1	85,4	1,0	2,2	0,0	0,3	0,46	3,67	0,04	0,09	0,00	0,01	33,2	0,56
07048	4,49	7,78	8,8	0,377	48,5	1,14	23,46	792	1,9	11,3	83,9	1,9	1,6	0,7	0,5	0,49	3,79	0,08	0,07	0,03	0,02	35,5	0,62
00166	3,51	7,56	8,7	0,377	49,9	1,15	22,96	908	2,3	17,9	77,2	1,8	2,7	0,1	0,2	0,53	2,80	0,06	0,10	0,00	0,01	33,9	0,60
99174	4,18	7,76	8,7	0,393	50,7	1,13	22,24	942	2,7	9,9	86,0	1,7	2,0	0,1	0,4	0,42	3,59	0,07	0,08	0,00	0,02	31,9	0,67
03103	3,26	7,87	8,8	0,390	49,6	1,12	22,60	909	2,6	12,7	83,0	1,6	2,5	0,0	0,3	0,42	2,70	0,05	0,08	0,00	0,01	34,1	0,53
05097	3,47	7,15	8,1	0,358	50,1	1,13	22,48	920	4,6	14,0	82,0	1,1	2,5	0,1	0,4	0,51	2,81	0,04	0,09	0,00	0,01	31,0	0,63
08019	3,06	7,47	8,8	0,386	51,8	1,18	22,79	913	1,9	9,9	86,7	1,0	2,0	0,1	0,3	0,30	2,66	0,03	0,06	0,00	0,01	35,3	0,52
08053	3,86	7,52	8,7	0,383	51,0	1,16	22,65	859	2,2	17,2	78,8	1,8	1,7	0,1	0,5	0,64	3,06	0,07	0,06	0,00	0,02	33,8	0,68
08054	4,59	7,69	8,6	0,386	50,3	1,12	22,18	908	2,7	14,0	81,7	1,5	2,2	0,1	0,5	0,64	3,76	0,07	0,10	0,00	0,02	33,3	0,55
09005	4,43	7,90	9,0	0,405	51,3	1,14	22,14	926	1,9	9,5	86,8	1,4	1,8	0,1	0,4	0,41	3,85	0,06	0,08	0,00	0,02	35,4	0,67
05104	4,18	8,12	8,8	0,393	48,5	1,09	22,38	891	2,0	14,8	80,4	1,4	3,0	0,0	0,4	0,59	3,39	0,06	0,12	0,00	0,02	31,5	0,64
08044	5,08	7,94	8,6	0,386	48,7	1,09	22,30	993	2,4	14,3	81,9	1,5	1,9	0,1	0,4	0,69	4,20	0,07	0,09	0,00	0,02	33,5	0,58
07132	3,47	7,37	8,8	0,391	53,1	1,19	22,52	952	2,4	11,7	84,7	1,6	1,4	0,2	0,4	0,40	2,95	0,05	0,05	0,01	0,01	32,5	0,66
07134	4,47	7,39	8,8	0,383	51,7	1,19	22,93	945	2,2	15,0	81,4	1,4	1,3	0,1	0,7	0,66	3,64	0,07	0,06	0,01	0,03	34,6	0,59
07136	3,93	7,56	8,7	0,394	52,1	1,15	22,04	901	2,0	10,7	86,6	1,1	1,0	0,1	0,4	0,42	3,41	0,05	0,04	0,00	0,02	35,0	0,80
09033	4,41	7,74	9,0	0,387	49,9	1,16	23,29	1022	1,9	17,7	78,4	1,6	1,6	0,1	0,6	0,69	3,55	0,07	0,07	0,00	0,03	32,8	0,66
09042	3,89	7,20	8,4	0,378	52,5	1,16	22,15	872	2,6	14,8	82,4	1,1	1,2	0,1	0,4	0,56	3,22	0,04	0,05	0,01	0,02	34,5	0,66
08096	3,91	7,54	8,7	0,387	51,3	1,15	22,48	779	1,8	9,9	86,5	1,3	1,9	0,1	0,4	0,38	3,39	0,05	0,07	0,00	0,01	33,3	0,68
09059	3,79	7,85	8,9	0,400	50,9	1,13	22,16	831	1,6	11,0	85,8	1,2	1,5	0,1	0,4	0,42	3,26	0,05	0,05	0,00	0,02	34,7	0,51
03S002	4,24	7,55	8,8	0,385	51,0	1,17	23,01	911	2,0	10,5	85,4	1,4	2,1	0,1	0,5	0,44	3,62	0,06	0,08	0,00	0,02	35,4	0,58
04S003	4,29	7,47	8,9	0,390	52,2	1,19	22,76	868	2,0	10,6	85,7	1,7	1,4	0,1	0,6	0,43	3,71	0,07	0,05	0,01	0,03	36,7	0,52
04S004	3,14	7,49	8,6	0,383	51,2	1,16	22,59	851	3,0	19,5	76,3	1,6	2,0	0,1	0,6	0,65	2,35	0,05	0,06	0,00	0,02	32,6	0,61
03100	3,11	7,64	8,8	0,383	50,2	1,15	22,90	885	2,2	13,3	82,7	1,2	2,5	0,0	0,3	0,40	2,58	0,04	0,08	0,00	0,01		0,59
06106	3,32	8,06	8,8	0,398	49,4	1,10	22,22	906	2,1	18,0	77,5	1,5	2,3	0,1	0,5	0,57	2,61	0,05	0,07	0,00	0,02	30,3	0,63
08093	3,86	7,62	8,6	0,389	51,1	1,13	22,09	990	2,4	13,2	83,0	1,5	1,8	0,1	0,5	0,44	3,28	0,06	0,07	0,00	0,02	34,2	0,61
08094	4,10	7,49	8,5	0,380	50,8	1,13	22,34	918	2,7	9,8	87,0	1,2	1,4	0,1	0,4	0,40	3,57	0,05	0,06	0,01	0,02	34,3	0,50
08095	4,77	7,38	8,6	0,384	52,1	1,17	22,49	888	2,1	12,0	84,9	1,0	1,6	0,1	0,3	0,49	4,14	0,05	0,07	0,01	0,01	33,1	0,56
09C006	3,10	7,63	9,0	0,393	51,6	1,18	22,90	877	1,8	12,5	82,8	1,8	2,4	0,0	0,5	0,38	2,58	0,06	0,07	0,00	0,01	33,5	0,54
09S003	4,35	7,82	9,1	0,405	51,9	1,17	22,49	866	2,0	10,7	85,3	1,5	1,9	0,1	0,5	0,46	3,71	0,07	0,08	0,00	0,02	34,3	0,60
07C013	4,92	7,71	8,8	0,391	50,8	1,14	22,40	918	2,7	11,3	85,1	1,5	1,4	0,1	0,6	0,52	4,22	0,08	0,07	0,01	0,03	32,4	0,55
07S008	3,72	7,63	8,9	0,386	50,7	1,16	22,96	886	2,6	11,1	85,7	1,3	1,4	0,1	0,4	0,37	3,23	0,05	0,05	0,00	0,02	32,3	0,67
09S029	3,84	7,32	8,3	0,389	53,2	1,13	21,33	794	2,2	13,5	82,9	1,5	1,6	0,1	0,3	0,47	3,24	0,06	0,06	0,01	0,01	34,9	0,57
09S030	5,03	7,60	8,5	0,397	52,3	1,11	21,29	824	2,2	12,2	83,8	1,5	1,8	0,2	0,5	0,52	4,33	0,07	0,08	0,01	0,03	32,1	0,64
06S006	4,28	7,62	8,3	0,396	52,1	1,09	20,99	878	2,7	14,5	81,9	1,5	1,7	0,1	0,4	0,61	3,51	0,06	0,07	0,00	0,02	35,4	0,46
	37	37	37	37	37	37	37	37	37	37	37	37	37	37	37	37	37	37	37	37	37	36	37
	4,02	7,60	8,7	0,386	50,9	1,15	22,55	894	2,3	12,7	83,4	1,4	1,9	0,1	0,4	0,49	3,38	0,06	0,07	0,00	0,02	33,7	0,60
	3,06	7,15	8,1	0,358	48,5	1,09	20,99	779	1,6	7,9	76,3	1,0	1,0	0,0	0,2	0,30	2,35	0,03	0,04	0,00	0,01	30,3	0,46
	5,08	8,12	9,1	0,405	53,2	1,22	24,34	1022	4,6	19,5	88,2	2,3	3,0	0,8	0,7	0,69	4,33	0,09	0,12	0,03	0,03	36,7	0,80

1) Source: All data were collected and archived at the test facility Experimental Toxicology and Ecology, BASF SE, 67056 Ludwigshafen, Germany, in accordance with the OECD principles of Good Laboratory Practice (GLP) and the GLP principles of the German "Chemikaliengesetz" (Chemicals Act)

HISTORICAL CON

Species:
Sex:
Strain:
Age:
Study period:
Fasting before bloo
Anaesthesia
Data print out at
Clinical Chemistry I
Haematology Analy
Coagulation Analyz

Parameter:	AST	ALP	SGGT	NA	K	CL	INP	CA	UREA	CREA	GLUC	TBIL	TPROT	ALB	GLOB	TRIG	CHOL
Unit:	MYKAT/L	MYKAT/L	NKAT/L	MMOL/L	MMOL/L	MMOL/L	MMOL/L	MMOL/L	MMOL/L	MYMOL/L	MMOL/L	MYMOL/	G/L	G/L	G/L	MMOL/L	MMOL/L
Study No,																	
07088	1,58	0,87	0	140,0	4,07	102,5	1,65	2,59	6,90	51,1	5,06	2,58	62,72	37,69	25,02	0,44	1,37
07080	1,69	1,16	0	139,7	3,98	101,9	1,76	2,53	7,02	50,6	5,33	2,27	60,57	36,87	23,70	0,33	1,00
07105	2,69	1,61	0	139,3	4,05	102,1	1,82	2,60	7,32	54,1	5,01	2,50	61,47	37,03	24,44	0,39	1,65
07104	1,85	1,23	1	141,0	4,04	104,0	1,84	2,64	7,36	52,5	5,35	2,88	63,63	38,74	24,89	0,35	1,48
07048	1,57	1,04	5	141,4	4,01	104,0	1,59	2,51	7,34	54,9	5,60	2,82	62,96	36,95	26,00	0,29	1,11
00166	1,72	1,11	0	139,5	3,99	103,2	1,53	2,58	7,06	54,0	5,19	2,58	62,70	38,12	24,58	0,41	1,34
99174	1,71	0,99	0	140,9	4,05	103,3	1,75	2,63	7,34	51,3	5,42	2,55	64,69	38,56	26,12	0,44	1,48
03103	1,61	0,96	0	141,6	4,07	104,6	1,63	2,63	6,95	51,0	5,32	2,58	63,63	37,78	25,85	0,37	1,44
05097	1,67	1,11	0	140,1	3,94	103,6	1,84	2,56	8,49	56,1	5,05	2,50	66,12	40,16	25,96	0,47	1,51
08019	1,62	1,73	0	139,9	3,83	102,3	1,56	2,48	6,98	51,0	5,62	2,35	62,93	38,67	24,26	0,40	1,08
08053	1,85	1,38	0	140,0	3,87	102,7	1,64	2,54	5,80	50,1	5,91	1,75	60,15	36,73	23,42	0,31	0,95
08054	1,48	1,30	0	138,9	3,85	101,9	1,70	2,53	7,65	51,6	6,23	2,02	58,73	36,47	22,26	0,33	1,47
09005	1,76	1,57	5	141,1	4,08	102,2	1,83	2,63	6,96	52,2	5,16	2,23	61,97	39,21	22,76	0,33	1,23
05104	1,79	0,85	6	143,2	4,39	103,7	1,69	2,59	7,62	53,3	5,09	2,58	64,12	38,48	25,64	0,40	1,96
08044	1,83	0,71	8	144,1	4,14	104,8	1,76	2,56	7,34	50,8	5,07	2,35	59,61	35,44	24,17	0,36	1,28
07132	1,92	1,80	0	142,0	4,04	100,1	1,93	2,62	6,26	50,4	4,84	2,36	63,90	39,12	24,78	0,46	1,60
07134	2,00	1,44	0	143,8	4,14	103,1	1,81	2,53	6,97	53,9	5,47	2,43	60,81	39,73	21,07	0,39	1,38
07136	1,74	1,97	2	143,8	4,25	102,6	1,61	2,49	6,48	51,9	6,65	2,13	60,33	38,82	21,51	0,46	1,10
09033	1,99	0,88	1	142,6	4,12	101,9	1,79	2,65	7,12	55,0	5,54	2,36	62,04	40,18	21,85	0,35	1,28
09042	1,97	1,45	3	144,3	4,06	104,1	1,75	2,51	6,32	50,3	5,32	2,00	62,65	39,00	23,65	0,33	0,97
08096	1,92	2,01	14	140,4	4,19	101,8	1,81	2,50	6,73	50,2	5,28	2,22	60,20	38,41	21,79	0,41	1,16
09059	2,25	1,17	5	140,5	4,19	102,8	1,52	2,48	6,57	53,3	4,97	2,12	61,36	38,15	23,21	0,33	1,26
03S002	1,80	1,39	6	140,7	3,90	100,8	1,62	2,53	6,69	51,2	5,13	2,07	61,63	39,34	22,29	0,31	1,13
04S003	1,64	1,29	4	143,1	4,07	103,5	1,54	2,48	6,24	52,2	5,38	1,51	62,48	38,55	23,93	0,27	1,10
04S004	2,03	1,47	6	142,1	4,06	101,3	1,58	2,43	7,00	51,0	4,07	1,99	62,20	39,14	23,07	0,29	1,16
03100	1,70	1,22	0	141,1	3,79	102,8	1,74	2,52	6,31	50,9	4,84	2,28	66,16	39,89	26,27	0,42	1,27
06106	1,83	1,00	6	141,4	4,41	102,9	2,01	2,57	6,73	54,9	4,15	1,83	64,31	38,36	25,95	0,35	1,78
08093	1,82	1,42	17	140,9	4,09	102,7	1,53	2,46	5,94	50,0	5,71	2,14	59,41	37,73	21,69	0,40	1,25
08094	1,86	1,06	4	142,4	4,15	103,2	1,54	2,46	5,87	51,8	5,26	2,37	60,14	38,57	21,58	0,30	1,25
08095	2,01	1,49	6	140,3	4,20	101,9	1,83	2,51	6,65	54,5	5,72	2,36	62,26	39,42	22,84	0,52	1,39
09C006	1,61	1,67	6	142,5	4,18	102,4	1,59	2,50	6,64	50,0	5,19	1,68	63,69	39,20	24,49	0,40	1,34
09S003	1,51	1,19	11	141,7	4,29	102,0	1,75	2,49	6,57	47,0	4,72	2,52	58,40	37,33	21,07	0,29	1,35
07C013	1,68	1,49	4	142,4	4,01	102,2	1,90	2,58	7,14	51,7	5,33	2,60	61,45	39,02	22,43	0,29	1,52
07S008	1,88	1,40	6	143,2	4,15	103,4	1,58	2,53	6,66	50,6	5,96	2,23	61,05	39,04	22,01	0,30	1,77
09S029	1,65	1,30	11	141,4	4,17	102,8	1,71	2,58	6,92	51,8	6,72	2,14	61,55	40,28	21,27	0,31	1,22
09S030	1,85	1,43	13	141,1	4,16	101,8	1,64	2,55	6,96	49,3	6,13	2,01	62,01	39,80	22,21	0,41	1,34
06S006	1,36	1,12	1	142,7	4,15	102,3	1,61	2,51	5,74	48,7	5,68	2,17	62,20	40,66	21,53	0,41	1,37
	37	37	37	37	37	37	37	37	37	37	37	37	37	37	37	37	37
	1,80	1,30	4	141,5	4,08	103	1,70	2,54	6,83	51,76	5,36	2,27	62,06	38,56	23,50	0,37	1,33
	1,36	0,71	0	138,9	3,79	100	1,52	2,43	5,74	47,00	4,07	1,51	58,40	35,44	21,07	0,27	0,95
	2,69	2,01	17	144,3	4,41	105	2,01	2,65	8,49	56,10	6,72	2,88	66,16	40,66	26,27	0,52	1,96

1) Source: All data v
Toxicology and Eco
OECD principles of
German "Chemikali
