# Supplementary material for: Effects of SiO2, ZrO2, and BaSO4 nanomaterials with or without surface functionalization upon 28-day oral exposure to rats
Source: Arch Toxicol. 2014 Aug 28;88(10):1881–906. doi: 10.1007/s00204-014-1337-0 (PMC4161931; doi:10.1007/s00204-014-1337-0)
Supplement: Supplementary file 3 — Supplementary material 3 (PDF 47 kb) [file 204_2014_1337_MOESM3_ESM.pdf]

HISTORICAL CONTROL DATA OF CLINICAL PATHOLOGY TESTING [1)]

Species:		Rat
Sex:		Male
Strain:		Wistar
Age:		10 - 13 weeks
Study period:		4 weeks
Fasting before blood sampling:		16 hours
Anaesthesia		Isoflurane
Data print out at		5-Aug-14
Assay	Haptoglobin:	Immmunology Consultants Laboratory Inc., Newberg, OR, USA (cat. no. E-25HPT).
	alpha-2-macroglobulin:	Immmunology Consultants Laboratory Inc., Newberg, OR, USA (cat. no. E-25A2M).

Parameter:					Haptoglobin	A2M
Unit:					µg/ml	µg/mL
Study No.	srudy day	Animals examined	administration via	sampling month		
10I030	7	5	inhalation	Nov 2010	390,6	14,83
10I030	28	5	inhalation	Nov 2010	325,8	12,43
10I076	7	5	inhalation	Jan 2011	639,2	25,15
10I076	28	5	inhalation	Feb 2011	265,2	11,09
10I083	7	5	inhalation	Apr 2011	313,2	33,77
10I083	28	5	inhalation	Apr 2011	279,6	11,03
10I069	7	5	inhalation	Aug 2011	639,2	20,12
10I069	7	5	inhalation	Aug 2011	409,2	19,15
10I069	28	5	inhalation	Aug 2011	265,2	11,09
10I069	28	5	inhalation	Aug 2011	300,9	10,80
11I010	7	5	inhalation	Sep 2011	535,6	26,29
11I010	28	5	inhalation	Sep 2011	683,0	16,05
11I024	7	5	inhalation	Sep 2011	386,8	25,13
11I024	28	5	inhalation	Sep 2011	298,0	13,60
11I038	28	5	inhalation	Nov 2011	520,6	10,45
11I054	7	5	inhalation	Nov 2011	338,4	21,93
11I054	28	5	inhalation	Nov 2011	448,6	8,92
11I065	7	5	inhalation	Nov 2011	1022,2	13,58
11I065	28	5	inhalation	Nov 2011	1074,0	11,96
11I079	7	5	inhalation	Jan 2012	382,8	15,80
11I079	28	5	inhalation	Jan 2012	539,8	11,60
12I002	7	5	inhalation	Aug 2012	339,4	23,92
12I002	28	5	inhalation	Aug 2012	326,0	10,06
		N			23	23
		Mean			466,2	16,47
		Minimum			265,2	8,92
		Maximum			1074,0	33,77

1) Source: All data were collected and archived at the test facility Experimental Toxicology and Ecology, BASF SE, 67056 Ludwigshafen, Germany, in accordance with the OECD principles of Good Laboratory Practice (GLP) and the GLP principles of the German "Chemikaliengesetz" (Chemicals Act)
