# Supplementary material for: Effects of SiO2, ZrO2, and BaSO4 nanomaterials with or without surface functionalization upon 28-day oral exposure to rats
Source: Arch Toxicol. 2014 Aug 28;88(10):1881–906. doi: 10.1007/s00204-014-1337-0 (PMC4161931; doi:10.1007/s00204-014-1337-0)
Supplement: Supplementary file 4 — Supplementary material 4 (PDF 60 kb) [file 204_2014_1337_MOESM4_ESM.pdf]

HISTORICAL CONTROL DATA OF CLINICAL PATHOLOGY TESTING [1)]

Species: Rat
Sex: Male
Strain: Wistar
Age: 10 weeks
Study period: 4 weeks
Fasting before bloo 16 hours
Anaesthesia Isoflurane
Data print out at 5-Aug-14
Clinical Chemistry I Hitachi 917, Roche
Haematology Analy ADVIA 120, Bayer
Coagulation Analyz AMAX Destiny Plus, Trinity

Parameter:	WBC	RBC	HGB	HCT	MCV	MCH	MCHC	PLT	RETI	NEUT	LYMPH	MONO	EOS	BASO	LUC	NEUT	LYMPH	MONO	EOS	BASO	LUC	HQT	ALT
Unit:	GIGA/L	TERA/L	MMOL/L	L/L	FL	FMOL	MMOL/L	GIGA/L	%	%	%	%	%	%	%	GIGA/L	GIGA/L	GIGA/L	GIGA/L	GIGA/L	GIGA/L	SECONDS	MYKAT/L
Study No,																							
07088	6,92	7,65	9,0	0,384	50,2	1,18	23,53	821	1,5	12,5	82,3	2,0	1,8	0,9	0,6	0,86	5,70	0,14	0,12	0,06	0,04	38,5	0,76
07080	6,31	7,60	8,9	0,390	51,4	1,16	22,67	791	1,8	13,0	82,7	1,7	1,4	0,7	0,5	0,80	5,25	0,11	0,08	0,04	0,03	35,8	0,72
07105	5,81	8,00	9,5	0,404	50,5	1,18	23,45	921	2,0	14,0	81,6	1,5	2,6	0,0	0,3	0,80	4,75	0,09	0,15	0,00	0,02	36,1	0,79
07104	5,94	8,16	9,3	0,410	50,4	1,14	22,58	999	2,2	13,3	82,0	2,2	2,0	0,0	0,4	0,78	4,89	0,13	0,12	0,00	0,03	36,4	0,70
07048	7,84	7,95	9,2	0,400	50,3	1,16	23,09	826	1,8	11,0	84,9	1,7	1,1	0,7	0,6	0,84	6,66	0,14	0,09	0,05	0,05	34,9	0,78
00166	6,41	7,60	8,9	0,396	52,1	1,17	22,54	948	2,2	11,5	85,0	1,3	1,7	0,1	0,4	0,73	5,45	0,08	0,11	0,01	0,03	33,9	0,71
99174	5,49	8,18	9,2	0,415	50,7	1,13	22,20	947	2,3	15,4	80,8	1,4	2,1	0,1	0,3	0,83	4,45	0,08	0,11	0,00	0,02	34,3	0,74
03103	6,71	7,95	9,0	0,404	50,9	1,13	22,22	909	2,3	14,0	81,2	2,5	1,8	0,1	0,4	0,91	5,46	0,19	0,12	0,00	0,03	34,2	0,76
05097	5,17	7,85	9,4	0,415	52,8	1,19	22,55	896	2,2	13,5	82,9	1,3	1,9	0,0	0,3	0,70	4,27	0,07	0,10	0	0,02	36,6	0,89
08019	6,71	8,28	9,4	0,427	51,6	1,13	22,00	1006	2,2	9,80	86,5	1,3	2,1	0,1	0,3	0,64	5,82	0,09	0,14	0,00	0,02	37,1	0,68
08053	4,46	8,01	9,3	0,410	51,2	1,16	22,68	853	1,7	13,2	82,8	1,6	1,6	0,1	0,7	0,58	3,70	0,07	0,07	0,00	0,03	35,5	0,89
08054	7,07	8,16	9,2	0,415	50,9	1,13	22,16	910	2,1	13,8	81,9	1,5	2,2	0,1	0,5	0,93	5,83	0,10	0,15	0,01	0,03	36,7	0,78
09005	6,09	8,26	9,0	0,414	50,1	1,09	21,67	869	2,1	10,6	85,5	2,0	1,4	0,1	0,5	0,60	5,25	0,12	0,08	0,00	0,03	37,2	0,76
05104	6,16	8,46	9,0	0,407	48,1	1,06	22,11	853	2,0	14,8	80,0	1,9	2,8	0,1	0,5	0,91	4,94	0,11	0,17	0,01	0,03	34,9	0,77
08044	7,90	8,33	9,4	0,417	50,1	1,12	22,39	914	1,9	11,3	85,7	1,3	1,1	0,1	0,5	0,89	6,78	0,10	0,09	0,01	0,04	34,4	0,83
07132	4,52	8,47	9,5	0,432	51,1	1,12	22,00	1025	2,0	12,7	83,4	1,6	1,8	0,2	0,4	0,58	3,77	0,07	0,08	0,01	0,01	37,0	0,69
07134	5,09	7,72	9,2	0,406	52,7	1,19	22,68	880	1,9	13,8	82,1	2,0	1,4	0,1	0,7	0,69	4,19	0,10	0,07	0,01	0,04	37,6	0,77
07136	5,56	7,98	9,2	0,418	52,4	1,14	21,87	919	2,0	10,5	86,5	1,2	1,3	0,1	0,5	0,55	4,84	0,07	0,07	0,01	0,03	36,1	0,88
09033	6,80	8,05	9,3	0,406	50,5	1,15	22,87	1002	2,1	13,6	82,6	1,8	1,4	0,1	0,5	0,90	5,65	0,12	0,09	0,01	0,03	35,2	0,67
09042	4,38	7,64	9,0	0,407	53,3	1,17	22,04	909	1,8	14,2	82,7	1,4	1,1	0,1	0,5	0,60	3,65	0,06	0,05	0,01	0,02	37,6	0,80
08096	5,32	7,59	8,8	0,389	51,2	1,16	22,65	905	1,6	11,2	85,7	1,3	1,2	0,1	0,5	0,60	4,57	0,07	0,06	0,01	0,03	38,2	0,70
09059	6,95	7,89	9,1	0,419	53,2	1,16	21,81	917	2,0	11,0	85,7	1,4	1,2	0,1	0,5	0,74	5,98	0,10	0,09	0,01	0,03	38,4	0,68
03S002	5,32	7,97	9,2	0,416	52,2	1,16	22,23	962	1,9	13,3	82,6	1,8	1,6	0,1	0,7	0,72	4,38	0,09	0,08	0,00	0,04	37,2	0,67
04S003	4,82	8,11	9,1	0,409	50,4	1,12	22,33	952	2,1	12,4	83,6	1,5	1,6	0,1	0,7	0,60	4,03	0,07	0,08	0,01	0,03	36,9	0,58
04S004	5,21	7,84	8,8	0,400	50,9	1,12	22,04	1001	3,1	13,0	83,5	1,6	1,2	0,1	0,6	0,68	4,34	0,09	0,06	0,01	0,03	36,1	0,71
03100	5,30	8,02	9,1	0,405	50,6	1,13	22,40	887	2,1	12,9	83,1	1,5	2,2	0,1	0,3	0,66	4,43	0,08	0,11	0,00	0,02		0,74
06106	6,26	7,94	9,2	0,402	50,6	1,16	22,86	930	2,0	16,6	79,1	1,7	2,0	0,0	0,5	0,98	5,01	0,11	0,12	0,00	0,03	33,3	0,89
08093	5,79	8,21	9,3	0,420	51,2	1,13	22,04	978	2,0	13,8	81,6	2,1	1,7	0,2	0,6	0,77	4,76	0,12	0,10	0,01	0,03	35,5	0,79
08094	5,93	8,54	9,4	0,432	50,6	1,10	21,83	936	2,0	15,6	79,9	2,0	1,8	0,2	0,5	0,90	4,78	0,11	0,10	0,01	0,03	37,3	0,76
08095	6,00	8,10	9,4	0,425	52,5	1,16	22,08	933	1,4	10,8	85,8	1,6	1,2	0,1	0,6	0,65	5,15	0,10	0,07	0,01	0,03	34,8	0,68
09C006	5,68	7,88	9,2	0,414	52,7	1,17	22,23	872	1,9	11,7	83,7	2,1	1,6	0,1	0,8	0,62	4,80	0,12	0,08	0,01	0,04	38,6	0,80
09S003	7,37	8,60	9,5	0,429	49,9	1,11	22,24	934	2,1	12,6	83,2	1,7	1,8	0,1	0,6	0,90	6,17	0,12	0,13	0,01	0,05	38,3	0,63
07C013	5,71	7,87	9,2	0,412	52,4	1,17	22,39	960	2,8	9,4	87,0	1,4	1,5	0,1	0,6	0,53	4,97	0,08	0,08	0,01	0,03	36,8	0,81
07S008	5,76	7,76	9,0	0,404	52,1	1,16	22,27	883	2,4	12,4	83,8	1,8	1,4	0,1	0,5	0,70	4,86	0,09	0,08	0,01	0,03	34,3	0,68
09S029	6,26	7,87	8,6	0,415	52,8	1,10	20,80	857	2,3	12,0	85,0	1,4	1,2	0,2	0,3	0,73	5,35	0,08	0,07	0,01	0,02	36,1	0,78
09S030	5,72	8,10	8,8	0,428	52,9	1,08	20,43	849	2,4	10,7	86,5	0,9	1,3	0,1	0,5	0,60	4,96	0,05	0,07	0,01	0,03	34,0	0,67
06S006	6,00	8,13	8,7	0,425	52,3	1,07	20,50	875	2,3	14,2	81,4	2,3	1,7	0,1	0,5	0,80	4,94	0,13	0,09	0,00	0,03	39,6	0,53
	37	37	37	37	37	37	37	37	37	37	37	37	37	37	37	37	37	37	37	37	37	36	37
	5,97	8,02	9,1	0,411	51,3	1,14	22,23	914	2,1	12,7	83,4	1,7	1,6	0,2	0,5	0,74	4,99	0,10	0,10	0,01	0,03	36,3	0,74
	4,38	7,59	8,6	0,384	48,1	1,06	20,43	791	1,4	9,4	79,1	0,9	1,1	0,0	0,3	0,53	3,65	0,05	0,05	0,00	0,01	33,3	0,53
	7,90	8,60	9,5	0,432	53,3	1,19	23,53	1025	3,1	16,6	87,0	2,5	2,8	0,9	0,8	0,98	6,78	0,19	0,17	0,06	0,05	39,6	0,89

1) Source: All data were collected and archived at the test facility Experimental Toxicology and Ecology, BASF SE, 67056 Ludwigshafen, Germany, in accordance with the OECD principles of Good Laboratory Practice (GLP) and the GLP principles of the German "Chemikaliengesetz" (Chemicals Act)

HISTORICAL CON

Species:
Sex:
Strain:
Age:
Study period:
Fasting before bloo
Anaesthesia
Data print out at
Clinical Chemistry I
Haematology Analy
Coagulation Analyz

Parameter:	AST	ALP	SGGT	NA	K	CL	INP	CA	UREA	CREA	GLUC	TBIL	TPROT	ALB	GLOB	TRIG	CHOL
Unit:	MYKAT/L	MYKAT/L	NKAT/L	MMOL/L	MMOL/L	MMOL/L	MMOL/L	MMOL/L	MMOL/L	MYMOL/L	MMOL/L	MYMOL/	G/L	G/L	G/L	MMOL/L	MMOL/L
Study No,																	
07088	1,52	2,03	0	139,9	4,34	100,9	2,28	2,59	6,96	50,6	5,03	2,27	60,97	35,53	25,44	0,77	1,70
07080	1,55	1,98	0	141,3	4,67	102,2	2,13	2,59	5,89	45,8	5,64	2,06	60,40	35,78	24,62	0,78	1,69
07105	2,26	1,94	0	141,4	4,35	101,9	2,11	2,59	6,91	51,3	5,44	2,04	63,06	36,62	26,45	1,31	1,72
07104	1,82	2,04	0	143,4	4,58	104,0	2,22	2,70	6,55	50,2	5,89	2,32	62,06	37,19	24,87	0,92	1,59
07048	1,94	2,27	6	142,6	4,34	102,3	2,34	2,65	6,47	49,5	5,46	2,27	65,07	36,37	28,70	1,12	1,89
00166	1,72	2,02	0	139,9	4,47	100,8	2,11	2,56	6,22	46,9	5,92	2,37	62,09	37,06	25,03	0,81	1,79
99174	1,61	2,15	0	140,8	4,44	101,6	2,20	2,62	6,97	44,5	6,66	2,17	63,77	36,93	26,84	1,21	1,70
03103	1,71	2,00	13	141,0	4,59	101,7	2,27	2,65	6,50	46,7	5,75	2,63	63,93	37,15	26,78	0,88	1,72
05097	1,79	2,39	0	141,2	4,46	102,7	2,14	2,53	7,24	49,9	5,78	1,74	62,46	37,36	25,10	1,15	1,47
08019	1,86	2,77	0	140,5	4,51	100,4	2,17	2,57	7,28	48,2	6,37	2,12	64,31	37,97	26,33	0,89	1,49
08053	1,74	2,54	0	141,3	4,29	101,6	2,09	2,59	4,83	48,4	5,35	1,95	62,89	37,41	25,48	0,71	1,42
08054	1,81	1,82	0	139,1	4,33	100,0	2,14	2,60	7,08	50,7	5,49	2,25	61,61	37,14	24,47	0,94	1,55
09005	1,90	2,64	6	143,6	4,50	102,2	2,36	2,60	6,31	48,7	5,68	2,51	59,72	37,16	22,56	0,97	1,50
05104	1,87	1,50	6	144,4	4,72	103,3	2,09	2,59	6,97	48,2	5,13	2,35	61,70	36,20	25,50	0,88	2,15
08044	2,42	2,13	2	145,2	4,91	103,4	2,35	2,59	7,64	48,3	6,20	2,03	60,78	35,28	25,50	0,55	1,95
07132	1,90	2,45	0	143,4	4,42	100,9	2,08	2,60	7,67	51,1	6,53	2,13	63,12	38,21	24,91	0,80	1,44
07134	1,98	2,30	0	144,3	4,82	102,1	2,34	2,57	6,41	48,2	5,29	2,06	59,55	37,40	22,15	1,01	1,87
07136	1,84	2,15	1	143,6	4,66	100,9	1,93	2,52	6,34	47,6	7,60	1,93	59,94	36,45	23,48	1,35	2,12
09033	1,97	1,98	11	145,1	4,63	101,5	2,27	2,66	5,54	48,4	6,08	2,03	59,98	37,68	22,30	0,85	1,62
09042	1,86	2,77	0	146,0	4,45	103,1	2,16	2,57	4,69	43,9	5,52	1,88	63,36	38,34	25,01	0,55	1,41
08096	2,02	2,27	6	141,6	4,58	101,4	2,20	2,46	6,05	46,4	6,73	1,94	60,32	37,43	22,89	0,87	1,56
09059	1,92	2,27	1	141,4	4,75	100,6	2,22	2,54	6,73	48,7	5,94	1,94	61,16	37,27	23,88	0,87	1,57
03S002	1,46	2,26	5	142,7	4,58	101,0	1,88	2,60	6,23	47,3	5,75	1,85	63,37	38,19	25,18	0,93	1,60
04S003	1,73	2,17	2	143,9	4,49	101,6	2,05	2,47	5,97	48,1	6,10	1,46	60,78	36,55	24,23	0,85	1,41
04S004	2,41	2,59	3	143,6	4,68	99,7	2,14	2,42	6,31	46,7	3,88	1,97	61,39	37,67	23,72	0,82	1,60
03100	1,83	2,63	0	141,4	4,46	101,4	2,21	2,60	5,75	47,1	5,37	2,18	63,76	37,36	26,40	1,13	1,52
06106	1,74	2,38	0	140,5	4,53	102,4	2,34	2,59	5,90	47,3	4,97	1,87	62,79	36,95	25,84	0,69	1,92
08093	1,81	1,84	5	142,0	4,54	100,4	2,00	2,59	6,30	51,4	6,16	2,28	61,58	37,72	23,86	0,94	1,91
08094	1,84	1,85	8	142,2	4,68	101,5	1,97	2,57	6,47	50,3	6,70	1,81	60,84	37,33	23,51	1,19	1,79
08095	1,96	2,53	1	141,4	4,74	100,1	2,28	2,54	6,96	52,5	7,03	2,13	62,84	38,13	24,71	0,98	1,75
09C006	1,91	2,38	0	143,1	4,51	100,6	2,19	2,50	5,71	45,2	4,80	1,80	61,51	36,87	24,64	0,85	1,70
09S003	1,54	1,80	9	142,7	4,74	100,9	2,18	2,50	6,69	47,3	5,67	2,27	59,09	36,75	22,34	0,53	1,63
07C013	1,69	2,25	6	143,8	4,74	101,5	2,19	2,68	6,44	48,1	5,22	2,05	62,67	37,72	24,95	0,79	1,72
07S008	1,80	2,72	3	142,0	4,46	99,2	2,01	2,52	6,68	49,0	8,35	2,11	59,71	37,14	22,57	0,79	1,83
09S029	1,80	2,49	10	142,7	4,60	100,9	2,25	2,65	6,56	48,6	6,51	1,73	60,77	37,66	23,11	1,13	1,55
09S030	1,90	2,80	5	142,5	4,55	100,5	2,39	2,61	6,49	45,4	5,82	1,94	62,11	38,27	23,85	0,61	1,61
06S006	1,72	1,94	1	142,2	4,59	100,0	2,16	2,48	5,57	46,4	5,41	2,00	60,78	38,41	22,37	0,92	1,84
	37	37	37	37	37	37	37	37	37	37	37	37	37	37	37	37	37
	1,84	2,24	3	142,4	4,56	101,4	2,17	2,57	6,41	48,19	5,87	2,07	61,79	37,21	24,58	0,90	1,68
	1,46	1,50	0	139,1	4,29	99,2	1,88	2,42	4,69	43,90	3,88	1,46	59,09	35,28	22,15	0,53	1,41
	2,42	2,80	13	146,0	4,91	104,0	2,39	2,70	7,67	52,50	8,35	2,63	65,07	38,41	28,70	1,35	2,15

1) Source: All data v
Toxicology and Eco
OECD principles of
German "Chemikali
